# Balancing Food, Heat and Predation Risk: Seasonal and Diel Movement Strategies of African Herbivores

**DOI:** 10.1002/ece3.74498

**Published:** 2026-10-16

**Authors:** Zarah Schwan, W. Maartin Strauss, Johannes Signer, Robyn S. Hetem, Marco Heurich, Gaseitsiwe Masunga, Alan M. Wilson, Hattie L. A. Bartlam‐Brooks, Tatjana Y. Hubel, Emily Bennitt

**Affiliations:** ^1^ Okavango Research Institute University of Botswana Maun Botswana; ^2^ Applied Behavioural Ecology and Ecosystem Research Unit (ABEERU), Department of Environmental Sciences Florida Campus, University of South Africa Johannesburg South Africa; ^3^ School of Biological Sciences University of Canterbury Christchurch New Zealand; ^4^ Wildlife Sciences, Faculty of Forest Sciences and Forest Ecology University of Goettingen Göttingen Germany; ^5^ School of Animal, Plant and Environmental Sciences, Faculty of Science University of Witwatersrand Johannesburg South Africa; ^6^ Department of National Park Monitoring and Animal Management Bavarian Forest National Park Grafenau Germany; ^7^ Chair of Wildlife Ecology and Management, Faculty of Environment and Natural Resources University of Freiburg Freiburg Germany; ^8^ Department of Forestry and Wildlife Management University of Inland Norway Koppang Norway; ^9^ Structure and Motion Laboratory Royal Veterinary College, University of London Hatfield UK

**Keywords:** *Damaliscus lunatus*, *Equus quagga*, GPS telemetry, movement ecology, seasonal variation, tsessebe, ungulates, zebra

## Abstract

Understanding how large herbivores adjust their movement patterns in response to seasonal environmental variation is important for wildlife management. We investigated how sympatric African herbivores with contrasting body size, digestive systems and forage specialisation adjust their movement across seasons and the diel cycle. Using high‐resolution GPS data from 16 plains zebra ( *Equus quagga* ; hindgut fermenter) and 8 tsessebe ( *Damaliscus lunatus* ; ruminant) between 2014 and 2016 in the Okavango Delta, Botswana, we quantified 24 h range size, 24 h displacement distance, as well as displacement distance and tortuosity across 4 h time blocks, and evaluated these movements in response to season, maximum daily air temperature and vegetation greenness. Zebra occupied 24 h ranges double that of tsessebe during the rainy and early flood seasons, but 24 h range size converged during the late flood season. Zebra travelled twice the distance of tsessebe over 24 h during the early flood season, whereas travelling distances were similar during the rainy and late flood seasons. At finer temporal scales, both species showed pronounced but seasonally variable diel movement patterns. Movement paths were generally less tortuous in zebra than tsessebe, although interspecific differences diminished during the late flood season and midday. Movement patterns also changed with environmental conditions: increasing maximum daily air temperature was associated with a stronger reduction in movement in zebra than in tsessebe, whereas increasing vegetation productivity was associated with larger 24 h ranges and increased tortuosity, with the latter response stronger in zebra. Overall, our findings demonstrate that movement differences between zebra and tsessebe are strongly context‐dependent rather than constant across seasons. Movement convergence during the late flood season suggests that seasonal conditions can reduce otherwise pronounced interspecific differences in movement patterns. These findings improve our understanding of large herbivores' responses to seasonal environmental variation and may help predict species‐specific responses to future climate change.

## Graphical abstract

Using high‐resolution GPS data, we investigated how plains zebra and tsessebe in Botswana's Okavango Delta adjusted their movement across seasons, the diel cycle and changing maximum daily temperature and vegetation greenness. Zebra generally occupied larger ranges, travelled farther and followed less tortuous paths than tsessebe, although these differences varied with environmental conditions and diminished during late flood season and midday. Our findings demonstrate that movement differences between sympatric herbivores are strongly context‐dependent and may converge under particular seasonal conditions.

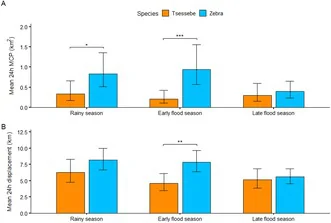

## 1 Introduction

In semi‐arid ecosystems, such as African savannas, rainfall‐driven seasonality alters both vegetation productivity and surface water availability, generating alternating periods of spatial constraints, when herbivores are forced to concentrate movements around limited water and forage, and spatial release, when widespread resource availability allows more flexible movement across the landscape (Redfern et al. 2003; Owen‐Smith and Mills 2008). The extent to which herbivores exploit these periods of spatial release varies among species, reflecting differences in digestive systems and forage selectivity, with more generalist grazers dispersing widely to track broadly distributed resources, while specialist grazers remain associated with a narrower set of productive habitats (Redfern et al. 2003; Owen‐Smith 2008; Fryxell et al. 2005). Outside the rainy season, limited surface water restricts many herbivores to areas near permanent water sources (Redfern et al. 2003; Valeix 2011), while declining soil moisture reduces grass quality and quantity (Smit 2011; Hassler et al. 2010), and predation risk may increase around those permanent water sources (Owen‐Smith and Mills 2008; Lone et al. 2017; Palmer et al. 2017; Morrison et al. 2016). In contrast, during the rainy season, surface water and high‐quality forage are widely available, allowing herbivores to disperse across the landscape (Hassler et al. 2010; Bartlam‐Brooks et al. 2011, 2013).

Movement enables herbivores to access spatially heterogeneous resources and allows individuals to adjust the amount and timing of foraging activity in response to temporal variation in resource availability (Goossens et al. 2020). At fine spatial scales, movement paths often mirror underlying resource distributions, with localised exploitation of high‐quality patches associated with more tortuous trajectories, whereas movement through lower‐quality resource landscapes tends to be more directed (Benhamou 2004; Bartumeus et al. 2005). Displacement distance provides an indication of the spatial extent of movement over a given period, with greater displacement generally reflecting increased movement activity or travel between resource patches (Street et al. 2018). Temporal variation in displacement and tortuosity can therefore yield valuable insights into how herbivores adjust their movement strategies to changing environmental conditions. Within seasons, short‐term variation in temperature and vegetation conditions may further influence movement behaviour, as herbivores can adjust the timing, extent and intensity of their movements in response to thermal constraints and changes in resource availability (Owen‐Smith 1998; Haylock et al. 2026).

The timing and distribution of foraging activity differ among species, reflecting digestive and associated physiological constraints. Ruminants depend on high‐quality forage and must allow time for rumination (Owen‐Smith and Novellie 1982; Belovsky 1986; Redfern et al. 2003), whereas hindgut fermenters compensate for lower forage quality by foraging for longer periods and resting less, allowing greater temporal flexibility across the diel cycle (Owen‐Smith and Goodall 2014; Schwarm et al. 2009). As a result, hindgut fermenters are generally less constrained by thermal conditions than ruminants (Owen‐Smith and Goodall 2014), although their reliance on maintaining gut fill limits the potential to shift between diurnal and nocturnal foraging (Owen‐Smith and Goodall 2014; Traill et al. 2016). Consequently, ruminants may have a greater scope than hindgut fermenters to shift a larger proportion of their activity towards cooler periods of the day when thermal stress is high (Maloney et al. 2005; Boyers et al. 2019, 2021). Among African browsing ruminants, body size further modulates these patterns with smaller species able to meet their daily energetic requirements by concentrating foraging at night and during the cooler hours of the day, whereas larger species often forage throughout much of the daylight period to meet their energetic demands and process lower quality forage (du Toit and Yetman 2005). Consequently, large‐bodied herbivores may be more constrained by thermoregulatory limits on foraging time (Belovsky 1986; Owen‐Smith 1998; du Toit and Yetman 2005; Owen‐Smith and Goodall 2014; Traill et al. 2016). Digestive system differences, in combination with body size, are therefore associated with contrasting patterns of space use. Large‐bodied hindgut fermenters can maintain their intake across broader landscapes with spatially variable forage quality, whereas small‐bodied ruminant grazers concentrate their activity within more restricted areas where high‐quality forage is available (Bell 1971; Belovsky 1986; Owen‐Smith 2008; du Toit and Yetman 2005). However, large herbivores continue to select high‐quality forage when it is available, and body mass alone is not a consistent predictor of fibre‐digestion capacity (Arsenault and Owen‐Smith 2008; Müller et al. 2013; Steuer et al. 2014). The extent to which these differences are expressed may vary with environmental conditions and resource availability.

In hot, semi‐arid environments, thermoregulatory demands often require large herbivores to seek shade during the hottest parts of the day and shift their activity to cooler periods, such as early morning, late afternoon, or night (Hetem et al. 2012; Boyers et al. 2019; Scheibe et al. 2009). However, nocturnal foraging may increase exposure to predation risk, as large carnivores such as African lions ( *Panthera leo* ) are more active at night (Packer et al. 2011; Cozzi et al. 2012). As a result, ungulate activity commonly follows a crepuscular rhythm, with peaks at dawn and dusk when temperatures are lower and light levels still allow effective predator detection, while activity declines around midday when thermal constraints are greatest (Owen‐Smith and Goodall 2014).

This general pattern is also evident in the Okavango Delta, northern Botswana (Bennitt et al. 2024), a flood‐pulsed wetland within a semi‐arid climatic region with seasonal changes in surface water distribution, ambient temperature and forage availability (McCarthy et al. 2000; Wolski and Murray‐Hudson 2006; Ramberg et al. 2006). During the rainy season, ephemeral pans across the landscape are filled and annual grasses provide highly productive forage. The concurrent rainfall in Angola leads to a flood pulse that rises during the early flood season, corresponding with cooler temperatures, a reduction in forage availability and a progressive drying of ephemeral pans. The flood pulse recedes during the late flood season, when high temperatures lead to senescent vegetation and surface water is restricted to permanent water channels. Within this strongly seasonal landscape, we used five‐minute GPS fixes collected from plains zebra ( *Equus quagga* ; hereafter zebra) and tsessebe ( *Damaliscus lunatus* ) to investigate how species‐specific movement patterns varied among seasons and across the diel cycle. Zebra are generalist, large bodied (~300–350 kg) hindgut fermenters with relatively low digestive efficiency and high intake capacity, allowing them to exploit abundant, low‐quality forage and track broad‐scale resource availability (Bell 1971; Illius and Gordon 1992; Owen‐Smith 2008). Tsessebe are medium‐sized (~120–140 kg) ruminant grazers with high digestive efficiency and strong specialisation on short, high‐quality grasses (Skinner and Chimimba 2005; Fryxell et al. 2005). Based on these differences in digestive physiology, body size and forage specialisation, we tested the following predictions: (i) zebra would occupy larger 24 h ranges than tsessebe, with interspecific differences being greatest under relatively favourable environmental conditions, and diminishing as environmental constraints increased; (ii) zebra would exhibit larger 24 h displacement distance than tsessebe, with the magnitude of this difference likewise varying with environmental conditions, (iii) increasing daily maximum temperature would alter the diel distribution of movement, with zebra, as the large hindgut fermenter and therefore better able to tolerate lower quality forage, reducing their movement during the midday more than the small ruminant, tsessebe, particularly during the late flood season when heat loads are highest; and (iv) tsessebe movement paths would be more tortuous than those of zebra, with peaks in tortuosity during the night and cooler times of day, when foraging activity was expected to be greatest, particularly when vegetation greenness and maximum daily air temperatures were highest.

## 2 Methods

### 2.1 Study Area

The data were collected in the south‐eastern part of the Okavango Delta, a conservation area in northern Botswana, including the Moremi Game Reserve and wildlife management areas NG33 and NG34 (Figure 1). We distinguished three seasons based on rainfall and flood dynamics: the rainy season (November to April), when rainfall drives widespread forage growth; the early flood season (May to July), when floodwaters begin to rise and expand across the floodplains; and the late flood season (August to October), when floodwaters recede and surface water becomes increasingly confined to channels and permanent water bodies (McCarthy et al. 2000; Wolski and Murray‐Hudson 2006; Bennitt et al. 2024). Rainfall records from the study period confirmed that precipitation was concentrated between November and April in both study years, supporting the seasonal classification, although annual rainfall differed between years, with 306 mm recorded in 2014/2015 and 430 mm in 2015/2016 (Bennitt et al. 2019; Bartlam‐Brooks et al. 2013).

**FIGURE 1:**
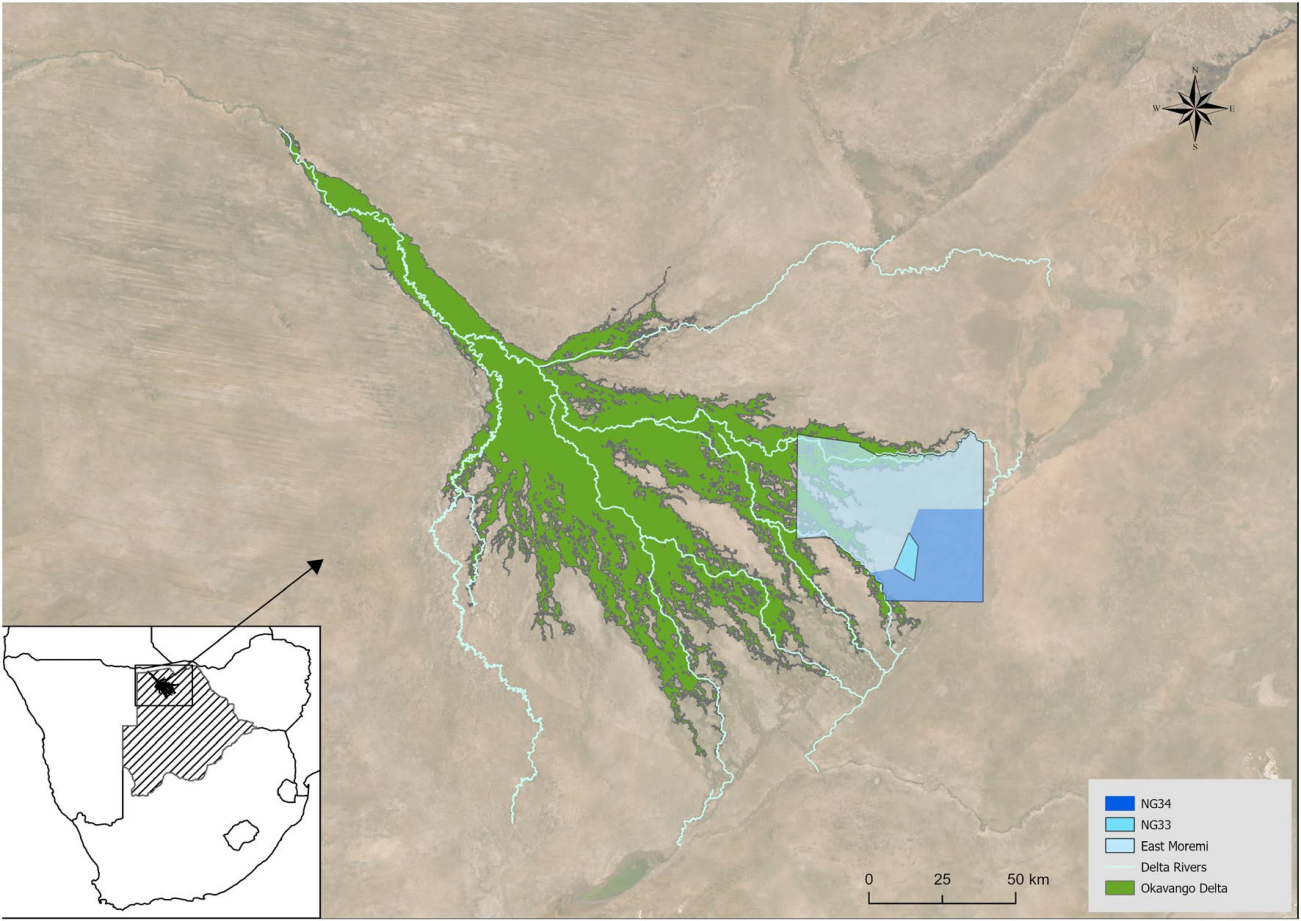
Location of the study area in the Okavango Delta, Botswana. Data were collected from zebra and tsessebe in the eastern part of the Moremi Game Reserve and the surrounding management areas NG33 and NG34 from 2014 to 2016.

During the rainy seasons in 2014–2016, mean daily maxima of air temperatures were ~31°C–33°C, while nighttime temperatures remained relatively warm at ~19°C–21°C. Conditions were considerably cooler during the early flood season, with maximum daytime air temperatures of ~25°C–28°C and nighttime lows of ~9°C–11°C. The late flood season was the hottest period of the year, with daytime temperatures of ~32°C–34°C and nighttime temperatures of ~16°C–18°C (Department of Meteorological Services, Botswana 2025). The study area was comprised of grassland, riparian woodland, mopane woodland and acacia woodland (Bennitt et al. 2014). The study area supported a diverse assemblage of large herbivore species, including tsessebe, zebra, southern giraffe ( *Giraffa giraffa* ), blue wildebeest ( *Connochaetes taurinus* ), greater kudu ( *Tragelaphus strepsiceros* ), impala ( *Aepyceros melampus* ), African elephant ( *Loxodonta africana* ) and Cape buffalo ( *Syncerus caffer* ), and five large carnivore species: African lion, African wild dog ( *Lycaon pictus* ), leopard (*Panthera pardus*), spotted hyaena ( *Crocuta crocuta* ) and cheetah ( *Acinonyx jubatus* ) (Bennitt et al. 2024).

### 2.2 Collars

For this study, eight adult female tsessebe and 16 adult female zebra were fitted with GPS collars programmed to record GPS fixes every 5 min. Collars were developed and manufactured by the Structure and Motion Laboratory of the Royal Veterinary College (Wilson et al. 2013), University of London; details of collar design can be found in Hubel et al. (2016), Wilson et al. (2013) and Wilson et al. (2018).

Females were chosen because they represent core breeding herds. All collared individuals belonged to separate groups. Seven zebra were collared in November 2014, one in September 2015 and eight in October 2015. All tsessebe were collared at the beginning of September 2015. All collars included an automatic drop‐off unit (Sirtrack, Hawkes Bay, New Zealand), which was programmed to trigger a year after collar fitting. All drop‐offs on zebra collars functioned as programmed, but three tsessebe had to be re‐darted at the end of the study to remove the collars because of drop‐off failure.

Tsessebe were immobilised with Thiafentanil (4 mg), Azaperone (80 mg), 1700 i.u. Hyalase and topped up post‐capture with Ketamine (100 mg; Bennitt et al. 2024). Zebra were immobilised with Etorphine (7 mg), Azaperone (80 mg), and 1700 i.u. Hyalase (Bennitt et al. 2024). All herbivores were reversed with Naltrexone as soon as collars had been fitted (Bennitt et al. 2024). Dart sites were injected with local antibiotics to prevent infection. Heavily pregnant animals and those with small young were avoided, and all individuals were observed rejoining their original groups following immobilisation and collar fitting.

All animals were darted by an experienced veterinarian registered in Botswana following the issuing of research permit EWT 8/36/4 XXIV (199) and associated supplementary permits by the Department of Wildlife and National Parks of Botswana (Bennitt et al. 2024). Methods were approved by the Ethics and Welfare Committee of the Royal Veterinary College, London (URN 20131233).

### 2.3 Data Analysis

All data processing and statistical analyses were conducted in R version 4.3.2 (R Core Team 2023). Each GPS fix was assigned to one of three seasons based on its recording date: rainy season (November–April), early flood season (May–July) and late flood season (August–October). To account for environmental conditions beyond seasonal classification, we included maximum daily air temperature and vegetation productivity as explanatory variables. Daily maximum air temperature was obtained from a weather station based in Maun (Botswana Meteorological Services) and assigned to each observation based on its recording date. Vegetation productivity was quantified using the Enhanced Vegetation Index (EVI) derived from the MODIS MOD13Q1 Version 6.1 product (250 m spatial resolution; 16‐day temporal resolution). Each observation was assigned the corresponding EVI value for the respective 16‐day composite period. Before statistical modelling, maximum daily air temperature and EVI were centred and scaled to improve model convergence. Collinearity among season, maximum daily air temperature and EVI was assessed using variance inflation factors (VIFs), with generalised VIFs adjusted for degrees of freedom used for the categorical season variable. Maximum daily air temperature and EVI were moderately negatively correlated (Pearson's *r* = −0.31), while all adjusted VID values were < 2, indicating low collinearity among predictors. Across analyses, seasonal and interspecific differences in movement were evaluated using linear mixed effects models (LMMs) implemented in the lme4 package (Bates et al. 2015). For each response variable, we specified a global model containing the explanatory variables and biologically relevant interactions appropriate to that analysis (Tables S1, S3, S5 and S8). Candidate predictors included species, season, time block (representing different periods of the diel cycle), maximum daily air temperature and EVI, with the exact model structure depending on the response variable. Individual identity was included as a random intercept to account for repeated measurements within individuals. Response variables were log‐transformed where necessary to improve conformity with model assumptions. Model assumptions were assessed using simulation‐based residual diagnostics implemented in the DHARMa package (Hartig 2026), including visual inspection of residual diagnostics and tests for dispersion. For each response variable, the global model was compared with a predefined set of simpler candidate models of each individual fixed effect, their interactions and combinations thereof (Tables S1, S3, S5 and S8). Models were compared using Akaike's Information Criterion corrected for small sample sizes (AICc; Akaike 1974). Following the information‐theoretic approach of Burnham and Anderson (2002), we ranked candidate models according to AICc and considered the model with the lowest AICc as the best‐supported model, while ΔAICc and Akaike weights were used to evaluate the relative support for competing models with the most parsimonious model selected. Estimated marginal means (EMMs) and pairwise contrasts were obtained for species, season, time block and their interactions, as appropriate for each best‐supported model, using the emmeans package (Lenth et al. 2025). Species differences were evaluated within seasons and seasonal differences within species using Tukey‐adjusted contrasts to control for multiple testing. Estimates were back‐transformed to the original scale for interpretation and presentation. Statistical significance of pairwise contrasts was assessed. Figures were generated using *ggplot2* (Wickham 2016).

#### 2.3.1 24 h Ranges

We extracted individual 24 h ranges from sunrise to sunrise and quantified their spatial extent using 100% Minimum Convex Polygons (MCPs) (Mohr 1947). Over such short time scales, utilisation distribution estimators (e.g., kernel density estimators or Brownian bridge movement models) are sensitive to autocorrelation, smoothing parameter selection and assumptions about range residency, potentially biassing estimates of space use (Worton 1989; Horne et al. 2007; Fieberg and Börger 2012; Noonan et al. 2019). In contrast, MCPs require minimal assumptions and provide a transparent and comparable envelope of space use across individuals and days (Fieberg and Börger 2012). We therefore interpret MCPs as daily spatial extents rather than probabilistic home ranges, while acknowledging their sensitivity to outliers and inclusion of unused space (Laver and Kelly 2008; Demma and Mech 2011; Getz et al. 2007). We modelled log‐transformed 24 h MCP area using an LMM with species (zebra, tsessebe), season (rainy, early flood, late flood), maximum daily air temperature, EVI as fixed effects, with interactions between species and season, species and maximum temperature, species and EVI and individual identity as a random intercept (Equation 1).

$$\begin{array}{c} \log\left(\mathrm{MCP}\;\text{area}\right)\sim \text{species}\;\times \text{season}\;+\text{species}\;\times \\ \;\text{maximum temperature}+\text{species}\;\times \;\;\mathrm{EVI}+\left(1|\text{individual}\;\mathrm{ID}\right) \end{array}$$

#### 2.3.2 24 h Displacement Distance

Movement tracks were constructed using the *amt* package (Signer et al. 2024). The 24 h displacement distance was quantified by summing the step lengths of all 5‐min intervals between sunrise and sunrise, thereby representing the cumulative distance travelled during 24 h. The global model included the fixed effects of species, season, maximum daily air temperature, Enhanced Vegetation Index (EVI), the interaction between species and season, species and maximum daily air temperature, and species and EVI and individual identity as a random intercept (Equation 2)

$$\begin{array}{c} \log\left(\text{displacement distance}\right)\sim \text{species}\times \text{season}+\text{species}\times \\ \;\text{maximum temperature}\;+\;\text{species}\times \mathrm{EVI}+\left(1\;|\;\text{individual}\;\mathrm{ID}\right) \end{array}$$

#### 2.3.3 Four Hourly Displacement Distance

Hours of the day were aggregated into six fixed 4 h time blocks representing different periods of the diel cycle, namely late night: 00:00–03:59, dawn: 04:00–07:59, morning: 08:00–11:59, midday: 12:00–15:59, dusk: 16:00–19:59, and early night: 20:00–23:59. Four hourly displacement distances were calculated by summing all 5‐min step‐lengths per time block for each calendar day and individual.

The global model included species, season, time block, maximum daily air temperature and Enhanced Vegetation Index (EVI) as fixed effects, the three‐way interaction among species, season and time block, the interaction between species and maximum daily air temperature, the interaction between species and EVI and individual identity as a random intercept (Equation 3).

$$\begin{array}{c} \log\;\left(\text{DDblock}\right)\sim \text{species}\;\times \;\text{season}\times \text{time block}+\text{species}\;\times \\ \;\text{maximum temperature}+\text{species}\;\times \;\mathrm{EVI}+\left(1|\text{individual}\;\mathrm{ID}\right) \end{array}$$

#### 2.3.4 Tortuosity

Movement metrics were derived from 5‐min GPS data for six four‐hour time blocks, namely late night: 00:00–03:59, dawn: 04:00–07:59, morning: 08:00–11:59, midday: 12:00–15:59, dusk: 16:00–19:59, and early night: 20:00–23:59. For each individual and time block, cumulative displacement (CHD block) was calculated as the sum of distances moved between consecutive 5‐min GPS fixes across the entire 4‐h period. Straight‐line displacement (SHD block) was calculated as the straight‐line distance between the first and the last GPS location within the same 4‐h period. The resulting values represented one observation per individual per date and time block. Movement tortuosity was quantified using a tortuosity‐based index calculated as:

$${\mathrm{TI}}_{\text{Block}}=1-\frac{{\mathrm{SHD}}_{\text{block}}}{{\mathrm{CHD}}_{\text{block}}}$$

where SHD_block_ is the straight‐line displacement and CHD_block_ the cumulative path length per time block, such that values approaching zero indicate straighter movement and higher values indicate more tortuous paths (Benhamou 2004).

Movement tortuosity values were analysed using linear mixed effects models. The global model included the fixed effects of species, season, time block, maximum daily air temperature, Enhanced Vegetation Index (EVI), the three‐way interaction among species, season and time block, the interaction between species and maximum daily air temperature, the interaction between species and EVI and individual identity as a random intercept (Equation 4).

$$\begin{array}{c} \text{tortuosity}\sim \text{species}\times \text{season}\times \text{time block}+\text{species}\times \\ \;\text{maximum temperature}+\text{species}\times \mathrm{EVI}\;\left(1\;\text{individual}\;\mathrm{ID}\right) \end{array}$$

## 3 Results

### 3.1 24 h Ranges

The 24 h ranges were influenced by EVI, and the interactions between species and season, and between species and maximum daily air temperature (Table S1). Tsessebe exhibited significantly smaller 24 h ranges during the early flood season than during both the rainy season and the late flood season, whereas no difference was detected between the rainy and late flood seasons (Figure 2A, Table 1). In zebra, 24 h ranges were approximately half as large during the late flood season as during both the rainy and early flood season, whereas no difference was detected between the rainy and early flood seasons (Figure 2A, Table 1). Zebra occupied 24 h ranges twice as large as those of tsessebe during the rainy and early flood seasons, whereas no interspecific difference was detected during the late flood season (Figure 2A, Table S2).

**FIGURE 2:**
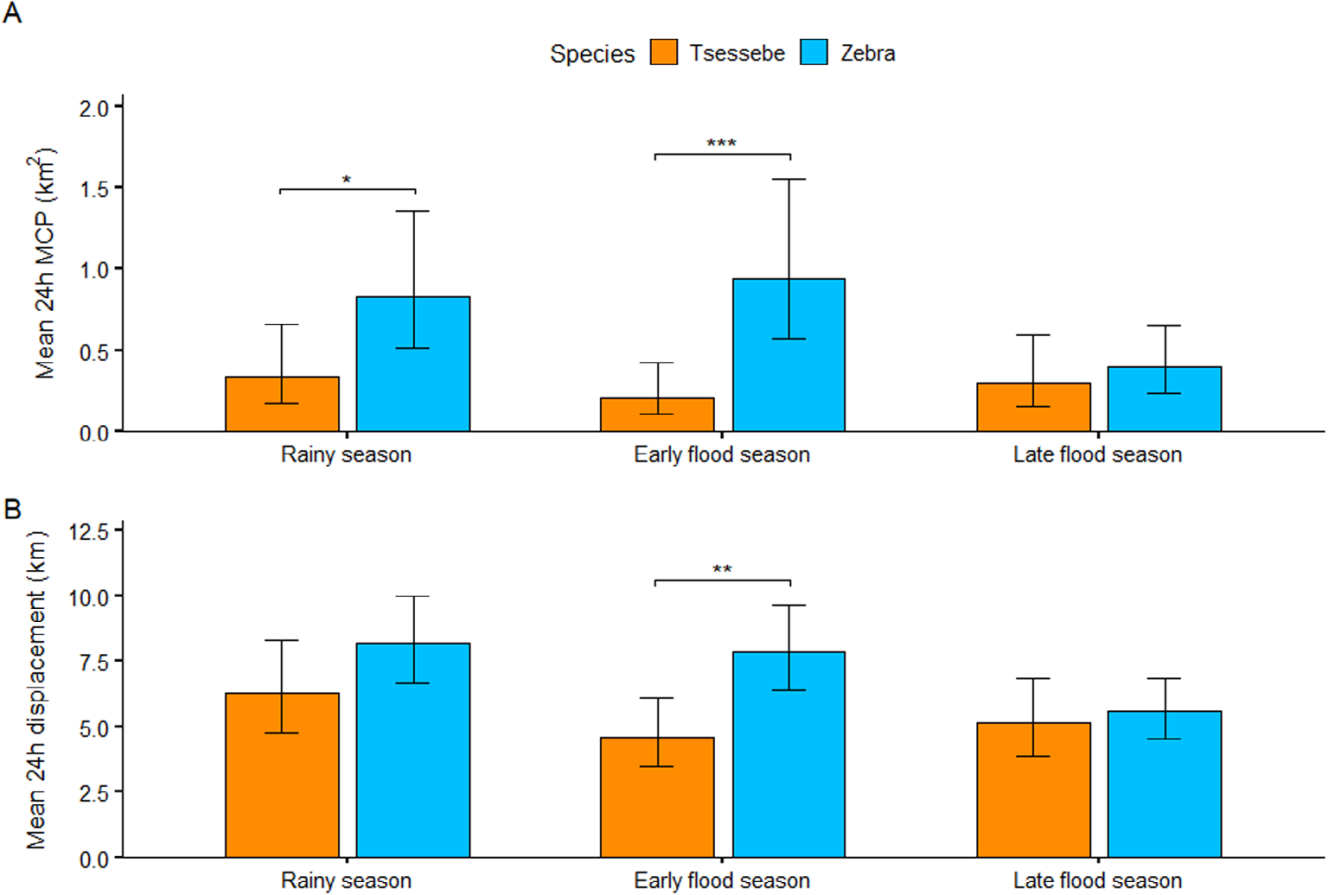
Estimated marginal means (±95% CI) of 24 h ranges (Minimum Convex Polygons, MCPs, in km2, panel A) and estimated marginal means of 24 h displacement distance (summed distance moved over 24 h, in km, panel B) of plains zebra (blue; *n* = 16) and tsessebe (orange; *n* = 8) across three seasons in the Okavango Delta, northern Botswana from 2014 to 2016. Values were derived from two separate linear mixed effects models (LMM) with log‐transformed MCP areas and 24 h displacement as response variables, species, season, maximum daily air temperature, Enhanced Vegetation Index (EVI) and the supported interaction terms as fixed effects, and individual ID as a random effect. Error bars represent 95% confidence intervals of the back transformed estimates. Horizontal lines indicate the pairwise comparisons associated with the significance annotations. Stars represent the interspecific difference with *** = *p* < 0.001, ** = *p* < 0.01 and * = *p* < 0.01 and * = *p* .

**TABLE 1:** Statistical results of Tukey‐adjusted post hoc comparisons testing intraspecific seasonal differences in 24 h range sizes (100% MCP; km^2^) of plains zebra and tsessebe in the Okavango Delta, northern Botswana from 2014 to 2016.

Species	Contrast seasons	Estimates	SE
Tsessebe	Rainy—Early flood	**0.46**	**0.11**
Tsessebe	Rainy—Late flood	0.10	0.10
Tsessebe	Early flood—Late flood	**−0.36**	**0.11**
Zebra	Rainy—Early flood	−0.12	0.09
Zebra	Rainy—Late flood	**0.75**	**0.10**
Zebra	Early flood—Late flood	**0.87**	**0.10**

*Note:* Estimates are presented on the log‐transformed scale of the fitted linear mixed‐effects model. Positive values indicate higher 24 h range sizes in the first‐listed season, whereas negative values indicate higher values in the second‐listed season. Bold numbers represent significance.

Maximum daily air temperature and EVI explained additional variation in 24 h range size. The relationship between 24 h range size and maximum daily air temperature differed between species, with zebra showing a stronger decline in range size with increasing temperature than tsessebe (Figure 3). In contrast, 24 h range size increased with increasing EVI across both species (Figure S1).

**FIGURE 3:**
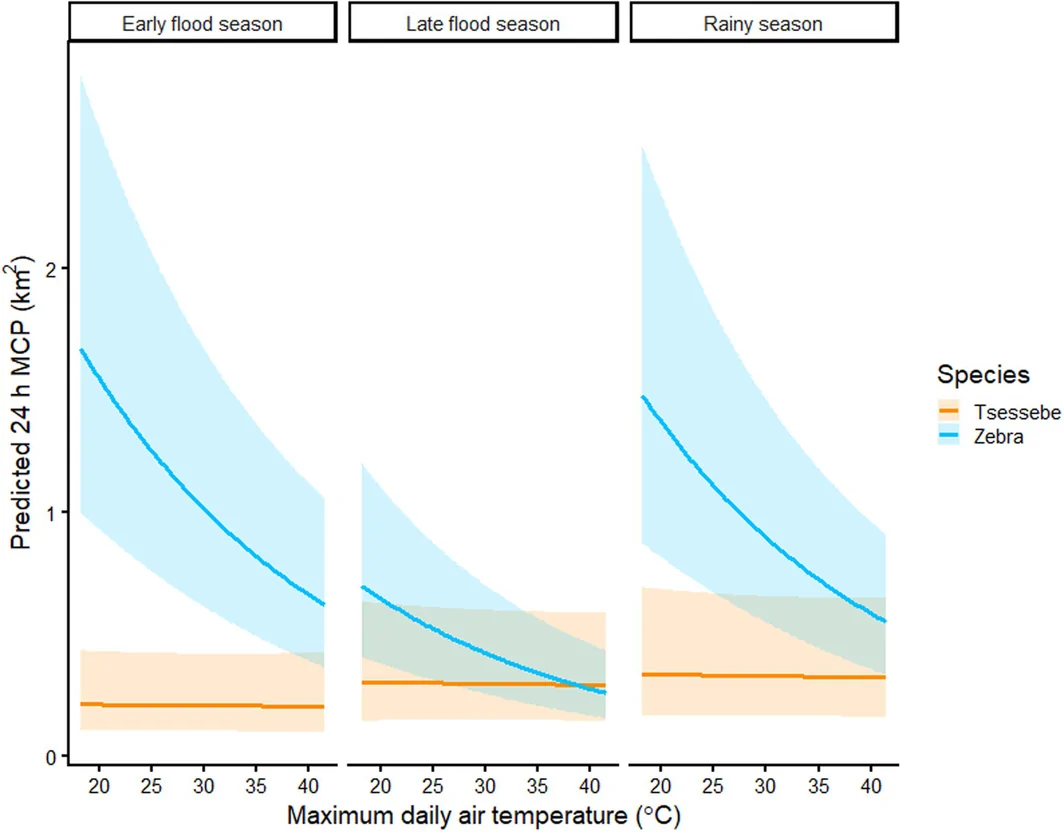
Predicted daily 24 h minimum convex polygon (MCP) range size (km2) of plains zebra (blue; *n* = 16) and tsessebe (orange; *n* = 8) across the observed range of maximum daily air temperature for the rainy, early flood and late flood seasons in the Okavango Delta, northern Botswana (2014–2016). Predictions were derived from the linear mixed‐effects model including species, season, maximum daily air temperature, EVI, the interaction between species and season and the interaction between species and maximum daily air temperature, with individual ID included as a random effect. Shaded areas represent 95% confidence intervals around the predicted values.

### 3.2 24 h Displacement Distance

The most parsimonious model for 24 h displacement included EVI, and the interactions between species and season, and between species and maximum daily air temperature (Table S3). Zebra 24 h displacements were similar in the early flood and rainy seasons, and reduced by a quarter in the late flood season (Tables 2 and S4, Figure 2B). Tsessebe 24 h displacements were highest in the rainy season, intermediate in the late flood season and smallest in the early flood season (Tables 2 and S4; Figure 2B). In the early flood season, zebra 24 h displacement was greater than that of tsessebe (Figure 2B); there was no difference in 24 h displacement between species in the late flood or rainy seasons (Tables 2 and S4; Figure 2B). Within each season, zebra exhibited a stronger decline in daily 24 h displacement with increasing maximum daily air temperature than tsessebe (Figure 4).

**TABLE 2:** Statistical results of Tukey‐adjusted post hoc comparisons testing intraspecific seasonal differences in 24 h displacement distance of plains zebra and tsessebe in the Okavango Delta, northern Botswana from 2014 to 2016. Comparisons were derived from the final linear mixed‐effects model after accounting for maximum daily air temperature and Enhanced Vegetation Index (EVI).

Species	Contrast seasons	Estimate	SE
Tsessebe	EarlyFlood—Late Flood	**−0.12**	**0.05**
Tsessebe	EarlyFlood—Rainy	**−0.31**	**0.04**
Tsessebe	Late Flood—Rainy	**−0.20**	**0.04**
Zebra	EarlyFlood—Late Flood	**0.34**	**0.04**
Zebra	EarlyFlood—Rainy	−0.04	0.04
Zebra	Late Flood—Rainy	**−0.38**	**0.04**

*Note:* Estimates are presented on the log‐transformed scale of the fitted model. Positive estimates indicate greater 24‐h displacement in the first‐listed season, whereas negative estimates indicate greater displacement in the second‐listed season. Bold numbers represent significance.

**FIGURE 4:**
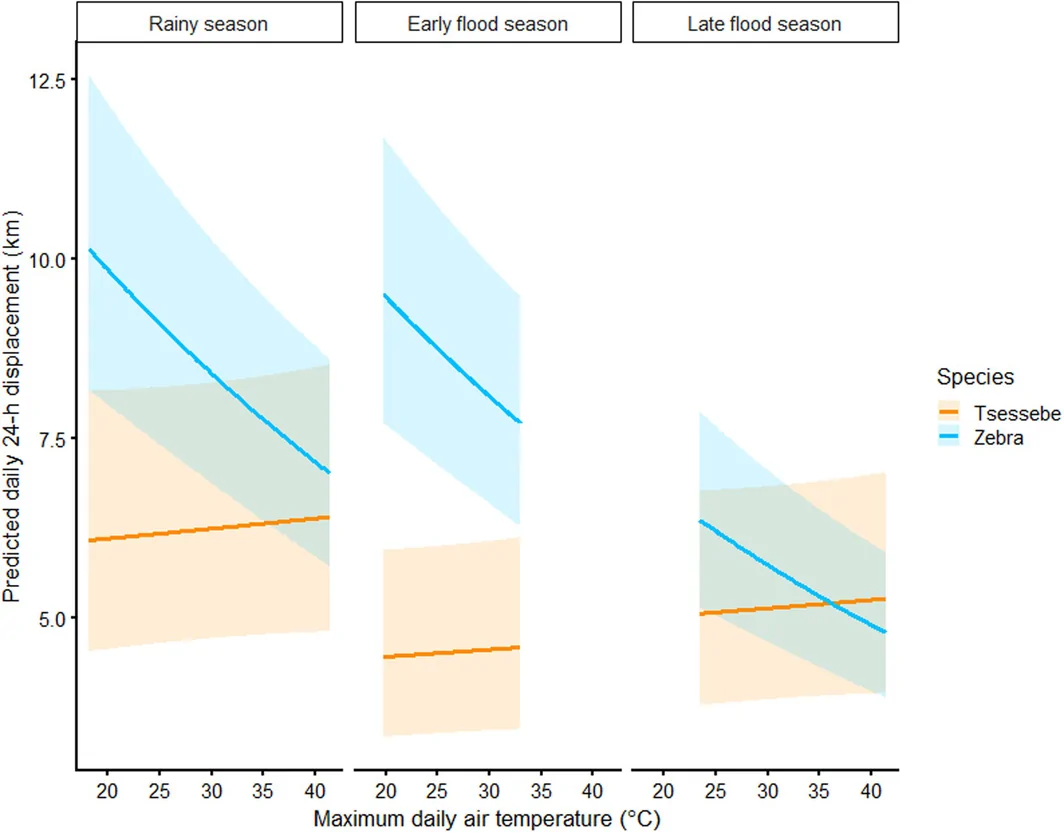
Predicted daily 24 h displacement distance (km) of plains zebra (blue; *n* = 16) and tsessebe (orange; *n* = 8) across the observed range of maximum daily air temperature for the rainy, early flood and late flood seasons in the Okavango Delta, northern Botswana (2014–2016). Predictions were derived from the final linear mixed‐effects model including species, season, maximum daily air temperature, EVI, the interaction between species and season and the interaction between species and maximum daily air temperature, with individual ID included as a random effect. Shaded areas represent 95% confidence intervals around the predicted values.

### 3.3 Four‐Hourly Displacement Distance

For the 4 h displacement distances, the best supported model included the three‐way interaction between species, season and time block, as well as an interaction between species and maximum daily air temperature, and between species and EVI (Table S5). The distance tsessebe travelled within 4 h was highest at dawn across seasons, followed by reduced movement in the morning. During the rainy and late flood seasons, displacement distance increased to a second peak at dusk; during the early flood season, this second peak occurred earlier, around midday. Four‐hourly displacement distance of zebra was highest at dusk during the rainy and late flood seasons, and at dawn during the early flood season. Zebra displacement distance decreased in the morning during the rainy and late flood seasons, but not during the early flood season (Figure 5). Four‐hourly displacement distance was lowest during the early and late night periods for both species (Figure 5, Table S6).

**FIGURE 5:**
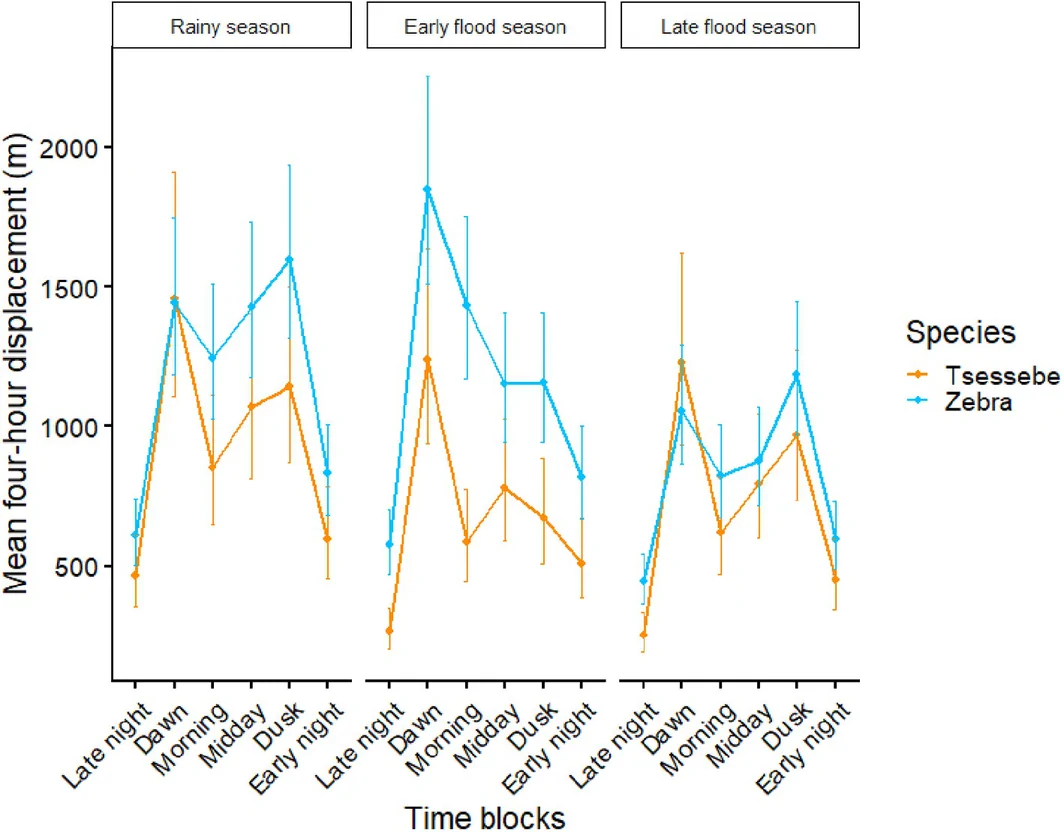
Estimated marginal means (±95% CI) of 4‐h displacement distance (cumulative hourly displacement, CHD; m) of plains zebra (blue; *n* = 16) and tsessebe (orange; *n* = 8) across six four‐hour time blocks (late night: 00:00–03:59; dawn: 04:00–07:59; morning: 08:00–11:59; midday: 12:00–15:59; dusk: 16:00–19:59; and early night: 20:00–23:59) and three seasons in the Okavango Delta, northern Botswana, from 2014 to 2016. Values were derived from the linear mixed‐effects model including species, season, time block, maximum daily air temperature, Enhanced Vegetation Index (EVI), the interaction among species, season and time block and the interaction between species and maximum daily air temperature, with individual ID included as a random effect. Error bars represent 95% confidence intervals of the back‐transformed estimated marginal means.

During the early flood season, zebra consistently moved more than tsessebe across all daytime blocks, as well as during the early and late night (Figure 5, Table 3). In the rainy season, zebra moved more than tsessebe in the morning, dusk and both early and late night blocks (Figure 5, Table 3). During the late flood season, zebra moved more than tsessebe only during the late night time block (Figure 5, Table 3).

**TABLE 3:** Interspecific differences in model‐estimated 4‐h displacement distance between plains zebra and tsessebe across six four‐hour time blocks and three seasons in the Okavango Delta, northern Botswana, from 2014 to 2016. Seasons comprised the early flood season (May–July), late flood season (August–October) and rainy season (November–April).

Season	Time block	Estimate	SE
Rainy	Dawn	0.01	0.17
Early flood	Dawn	**−0.40**	**0.18**
Late flood	Dawn	0.15	0.17
Rainy	Morning	**−0.38**	**0.17**
Early flood	Morning	**−0.89**	**0.18**
Late flood	Morning	−0.28	0.17
Rainy	Midday	−0.29	0.17
Early flood	Midday	**−0.39**	**0.18**
Late flood	Midday	−0.10	0.17
Rainy	Dusk	−0.33	0.17
Early flood	Dusk	**−0.54**	**0.18**
Late flood	Dusk	−0.20	0.17
Rainy	Early night	−0.33	0.17
Early flood	Early night	**−0.47**	**0.18**
Late flood	Early night	−0.28	0.17
Rainy	Late night	−0.27	0.17
Early flood	Late night	**−0.77**	**0.18**
Late flood	Late night	**−0.57**	**0.17**

*Note:* Values are presented as estimated marginal means derived from the final linear mixed‐effects model after accounting for maximum daily air temperature and Enhanced Vegetation Index (EVI). Estimates are presented on the log‐transformed scale of the fitted model, together with their standard errors. Tsessebe was used as the reference category; therefore, positive estimates indicate greater 4‐h displacement in zebra relative to tsessebe, whereas negative estimates indicate greater 4‐h displacement in tsessebe relative to zebra. Bold numbers represent significance.

Tsessebe generally moved more during the rainy season (Figure 5, Tables S6 and S7). Zebra were less consistent and moved farther during the midday in the rainy than early flood season; farther during dusk in the rainy than in the early flood season; and farther in the morning and dawn during the early flood than the rainy season (Figure 5, Tables S6 and S7).

Maximum daily air temperature and EVI explained additional variation in four‐hourly displacement. Across seasons and time blocks, zebra exhibited a significantly stronger decline in four‐hourly displacement distance with increasing maximum daily air temperature than tsessebe (Figure 6A). In contrast, four‐hourly displacement distance increased more strongly with EVI in zebra than in tsessebe (Figure 6B).

**FIGURE 6:**
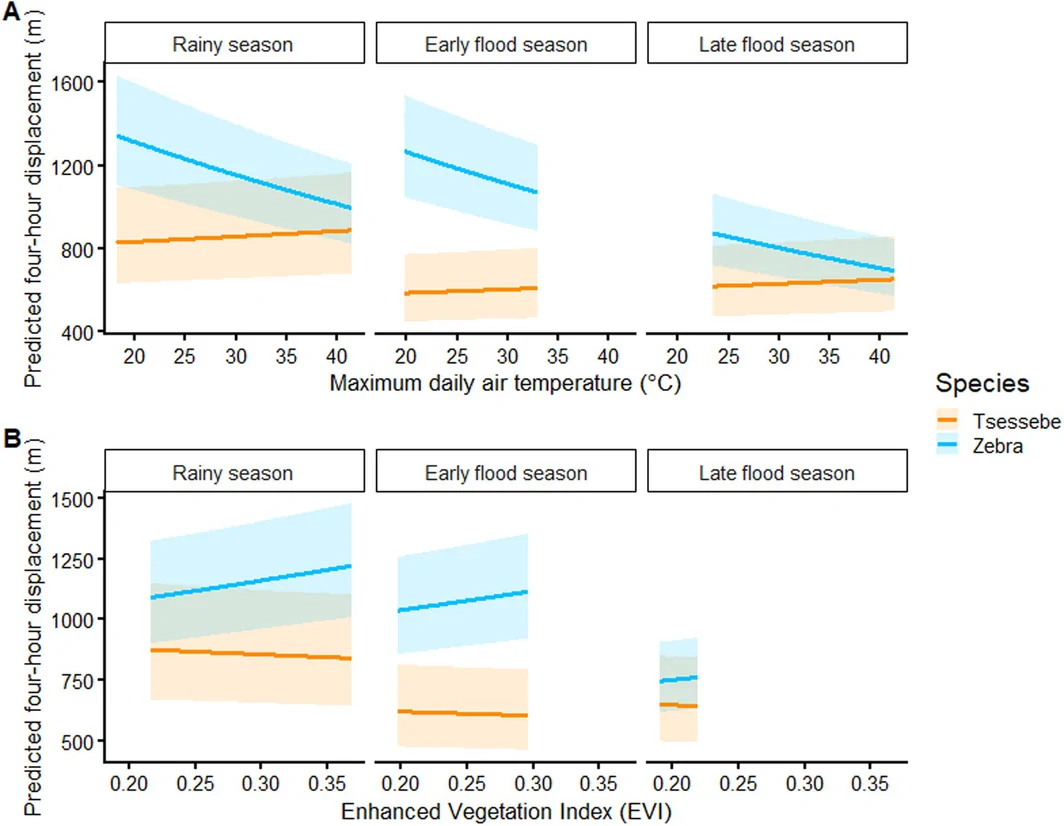
Predicted 4‐h displacement distance (CHD; m) of plains zebra (blue; *n* = 16) and tsessebe (orange; *n* = 8) in relation to maximum daily air temperature (panel A) and the Enhanced Vegetation Index (EVI; panel B) across the observed environmental conditions during the rainy, early flood and late flood seasons in the Okavango Delta, northern Botswana (2014–2016). Predictions were derived from the final linear mixed‐effects model including species, season, time block, maximum daily air temperature, Enhanced Vegetation Index (EVI), the three‐way interaction among species, season, and time block and the interaction between species and maximum daily air temperature, with individual ID included as a random effect. Lines represent model predictions, and shaded areas indicate 95% confidence intervals around the predicted values.

### 3.4 Tortuosity

Movement tortuosity was associated with the interactions among species, season and time block, and the interactions between species and both maximum daily air temperature and EVI (Table S8). Tortuosity values ranged between approximately 0.51 and 0.75, indicating moderately to highly sinuous movement paths (Figure 7). Tsessebe generally exhibited more tortuous movements than zebra across seasons, although species differences were less consistent during the late flood season (Figure 7, Table 4).

**FIGURE 7:**
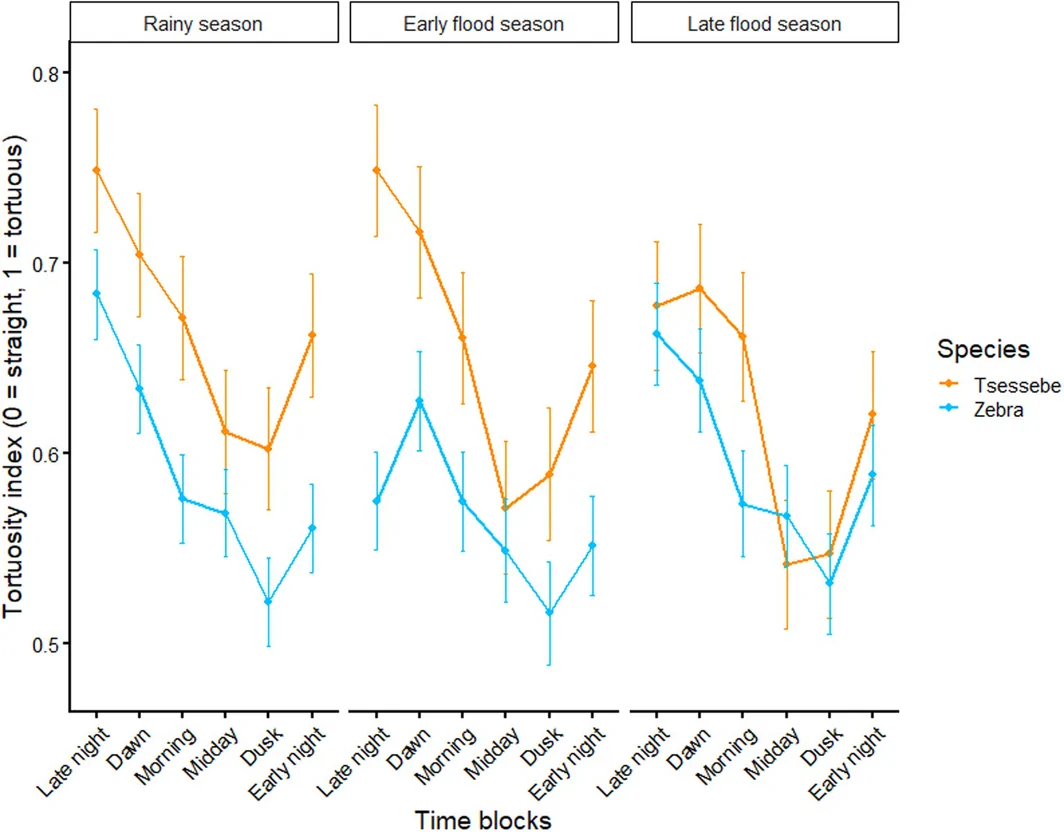
Estimated marginal means (±95% CI) of the tortuosity index of plains zebra (blue; *n* = 16) and tsessebe (orange; *n* = 8) across six four‐hour time blocks (late night: 00:00–03:59; dawn: 04:00–07:59; morning: 08:00–11:59; midday: 12:00–15:59; dusk: 16:00–19:59; and early night: 20:00–23:59) and three seasons in the Okavango Delta, northern Botswana, from 2014 to 2016. The tortuosity index was calculated as 1 − (Straight Hourly Distance/Cumulative Hourly Distance), where cumulative hourly distance (CHD) was calculated as the sum of distances moved between consecutive 5‐min GPS fixes within each hour and straight hourly distance (SHD) as the straight‐line distance between the first and last location within the same hour. Values were derived from the final linear mixed‐effects model including species, season, time block, maximum daily air temperature, Enhanced Vegetation Index (EVI), the three‐way interaction among species, season and time block, and the interaction between species and maximum daily air temperature, with individual ID included as a random effect. Error bars represent 95% confidence intervals of the estimated marginal means.

**TABLE 4:** Interspecific differences in model‐estimated tortuosity index between plains zebra and tsessebe across six four‐hour time blocks and three seasons in the Okavango Delta, northern Botswana, from 2014 to 2016. Seasons comprised the early flood season (May–July), late flood season (August–October) and rainy season (November–April).

Season	Time block	Estimate	SE
Rainy	Dawn	**0.07**	**0.02**
Early flood	Dawn	**0.089**	**0.02**
Late flood	Dawn	0.05	0.02
Rainy	Morning	**0.09**	**0.02**
Early flood	Morning	**0.09**	**0.2**
Late flood	Morning	**0.09**	**0.02**
Rainy	Midday	**0.04**	**0.02**
Early flood	Midday	0.02	0.02
Late flood	Midday	−0.02	0.02
Rainy	Dusk	**0.08**	**0.02**
Early flood	Dusk	**0.07**	**0.02**
Late flood	Dusk	0.02	0.02
Rainy	Early night	**0.10**	**0.02**
Early flood	Early night	**0.1**	**0.02**
Late flood	Early night	0.03	0.02
Rainy	Late night	**0.07**	**0.02**
Early flood	Late night	**0.17**	**0.02**
Late flood	Late night	0.01	0.02

*Note:* Values are presented as estimated marginal means derived from the final linear mixed‐effects model after accounting for maximum daily air temperature and Enhanced Vegetation Index (EVI). Estimates are presented on the fitted model scale, together with their standard errors, z‐ratios and adjusted *p*‐values. Tsessebe was used as the reference category; therefore, positive estimates indicate higher tortuosity in zebra relative to tsessebe, whereas negative estimates indicate higher tortuosity in tsessebe relative to zebra. Bold numbers represent significance.

Tsessebe showed significantly higher tortuosity than zebra during dawn, morning, dusk, early night and late night in the rainy and early flood seasons, as well as during the midday in the rainy season. During the late flood season, tsessebe showed significantly higher tortuosity than zebra during dawn and morning, whereas no significant species differences occurred during the midday, dusk, early night, or late night (Figure 7, Table 4). The largest species differences occurred during the late night in the early flood season (Figure 7, Table 4).

In tsessebe, tortuosity was highest during the late night in the rainy and early flood seasons and during dawn in the late flood season, whereas the lowest tortuosity occurred during dusk in the rainy and early flood seasons and during the midday and dusk in the late flood season (Figure 7). In zebra, tortuosity was highest during the late night in the rainy and late flood seasons and during dawn in the early flood season, whereas lower tortuosity generally occurred during dusk across all seasons (Figure 7, Tables S9 and S10). Species differences were generally smallest during the midday and were particularly reduced during the late flood season (Figure 7, Tables S9 and S10).

Across seasons and time blocks, movement tortuosity increased with increasing maximum daily air temperature in both species, particularly for tsessebe (Figure 8A). Similarly, movement tortuosity increased with increasing EVI in both species, but zebra responded more strongly to changes in EVI than tsessebe (Figure 8B).

**FIGURE 8:**
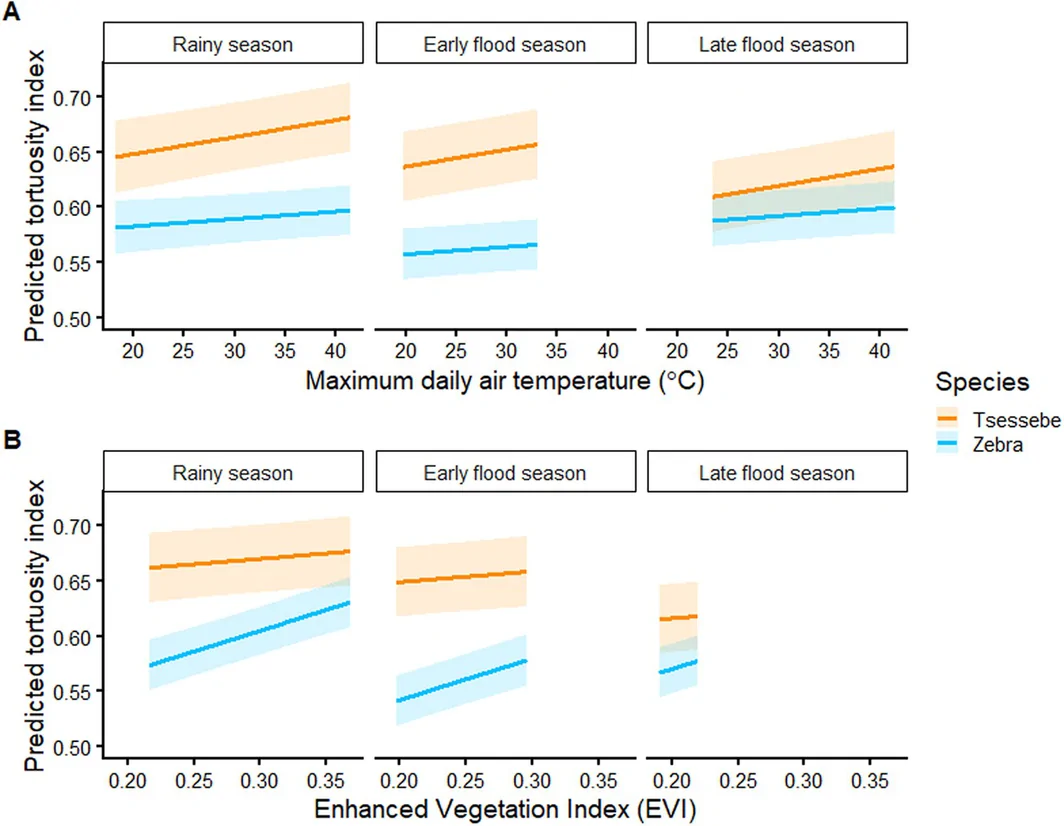
Predicted tortuosity index of plains zebra (blue; *n* = 16) and tsessebe (orange; *n* = 8) in relation to maximum daily air temperature (panel A) and the Enhanced Vegetation Index (EVI; panel B) across the observed environmental conditions during the rainy, early flood and late flood seasons in the Okavango Delta, northern Botswana (2014–2016). Predictions were derived from the final linear mixed‐effects model including species, season, time block, maximum daily air temperature, Enhanced Vegetation Index (EVI), the three‐way interaction among species, season, and time block and the interaction between species and maximum daily air temperature, with individual ID included as a random effect. Lines represent model predictions and shaded areas indicate 95% confidence intervals around the predicted values.

## 4 Discussion

Our findings demonstrate that zebra and tsessebe adjusted their movement patterns differently across seasons and throughout the day, with movement also varying in relation to temperature and vegetation greenness, indicating species‐specific responses to changing environmental conditions. Zebra 24 h ranges were twice as large as those of tsessebe in the rainy season and early flood season, but there was no difference in size during the late flood season, providing partial support for our prediction that zebra would use larger 24 h ranges than tsessebe and that interspecific differences would vary with environmental conditions. The 24 h ranges also increased with vegetation greenness, while increasing maximum daily air temperature was associated with a stronger reduction in range size in zebra than in tsessebe. Zebra exhibited greater 24 h displacement than tsessebe only in the early flood season. At a finer temporal resolution, both species showed pronounced diel structuring of movement, with displacement varying between species across time of day and season. Zebra generally moved farther than tsessebe, particularly during the day time blocks within the early flood season. Perhaps linked to the higher day time block displacement distances, zebra were more sensitive to changes in maximum daily air temperatures and EVI than tsessebe. Finally, tsessebe movement paths were more tortuous than those of zebra, particularly during the night and crepuscular time blocks during the rainy and early flood seasons, supporting our prediction of greater path tortuosity in the more specialised grazer during cooler times of day. Interspecific differences were generally smallest during the hottest period of the day, potentially reflecting reduced movement and shade‐seeking by both species, and were particularly reduced during the late flood season. In both species, tortuosity increased with vegetation greenness, although more strongly in zebra, potentially reflecting more localised exploitation of greener, high‐quality forage patches rather than unselective feeding across the landscape. Tortuosity also increased in both species with increasing maximum daily air temperature, although more strongly in tsessebe. Together, these results show that species differences in movement were strongly context‐dependent, with zebra generally using larger areas and moving farther, but these differences diminished or disappeared under late‐flood conditions. The contrasting fine‐scale responses to temperature and vegetation productivity further suggest that the two species differ in their behavioural responses to environmental variation. These differences may reflect their contrasting body sizes, forage specialisation and digestive strategies, although the effects of these traits cannot be disentangled in a two‐species comparison.

Zebra occupied twice as large 24 h ranges than tsessebe during the rainy and early flood seasons, consistent with their broader foraging strategy and the greater reliance on high intake rates to compensate for lower digestive efficiency (Owen‐Smith and Goodall 2014; Schwarm et al. 2009). The positive relationship between 24 h range size and EVI across both species suggests that greater vegetation productivity facilitated broader space use, potentially by increasing the spatial availability in forage. However, seasonal changes in other environmental factors, such as surface water availability, may also have contributed to these movement patterns. The smaller ranges occupied by tsessebe may have been linked to their more selective use of locally suitable forage patches (Bell 1971; Jarman 1974; Owen‐Smith 1998; Redfern et al. 2003). However, 24 h displacement was approximately twice as high in zebra as in tsessebe during the early flood season. During this period, floodwaters progressively expand across the Delta while many ephemeral rain‐fed pans begin to dry, resulting in a shifting mosaic of inundated and dry habitats. Consequently, suitable grazing areas on dry land become increasingly fragmented and redistributed, potentially requiring zebra to move greater distances between the remaining forage patches (McCarthy et al. 2000; Wolski and Murray‐Hudson 2006). In contrast, no interspecific differences in 24 h displacement were detected during the rainy or late flood seasons, despite differences in 24 h range size during the rainy season. This decoupling of daily spatial extent and moved distance suggests that tsessebe were concentrating movement within smaller areas, whereas zebra were distributing their movements across broader spatial footprints during the rainy and early flood seasons. Interspecific differences in 24 h range size and 24 h displacement were not apparent during the late flood season, suggesting a seasonal convergence in movement and space‐use under late flood conditions.

The 24 h displacement was relatively high in both species during the rainy season, consistent with increased movement under favourable environmental conditions, potentially reflecting responses to newly available forage, changes in surface water availability, or a combination of both (Gureja and Owen‐Smith 2002; Hempson et al. 2022). However, movement responses to seasonal patterns differed between species with zebra travelling the least per day during the late flood season, possibly reflecting species‐specific responses to seasonal changes in resource distribution and environmental conditions. (Smit 2011; Hempson et al. 2022). Together, these patterns highlight the importance of resource distribution and productivity in determining seasonal movement in large herbivores particularly when environmental constraints vary across seasons (Martin et al. 2018).

At finer temporal scales, movement metrics in both species showed pronounced diel patterns, which varied between species across seasons. Periods of greater displacement were generally associated with lower tortuosity, suggesting more directed movements that may reflect travel between resource patches. Indeed, zebra can use diel shifts between productive foraging areas and safer habitats to balance resource acquisition and predation risk (e.g., Courbin et al. 2019), however habitat use and predation risk were not directly quantified in our study. Periods of shorter movement distances and greater tortuosity, particularly during the late night and morning, are consistent with more localised activity and could reflect intensive use of forage patches. However, without concurrent behavioural observations, these movement patterns cannot be unequivocally attributed to foraging. Differences in digestive physiology may nevertheless contribute to the observed diel patterns, with tsessebe requiring periods of rumination and zebra relying on relatively continuous intake to maintain gut fill (Hofmann 1989; Owen‐Smith and Goodall 2014). Reduced movement during midday and night periods may similarly reflect reduced activity, resting, or rumination, but these behaviours cannot be distinguished from movement data alone. Similar circadian rhythms are widely reported across ungulates (Owen‐Smith and Goodall 2014; Courbin et al. 2019), including gemsbok ( *Oryx gazella* ) in arid systems (Boyers et al. 2021), and likely reflect trade‐offs among resource acquisition, thermoregulation and predation risk (Polansky et al. 2013; Owen‐Smith and Goodall 2014; Boyers et al. 2021).

During the late flood in the Okavango Delta system, receding floodwaters reduce the availability of productive floodplain habitat and ephemeral water bodies dry, potentially concentrating productive grazing areas and reliable water sources around floodplain margins and permanent channels. As grazing areas and reliable water sources concentrated, movement patterns between our herbivores converged, consistent with more localised movement rather than long‐distance travel, supporting the idea that seasonal resource constraints can reduce differences in space‐use strategies between species (van Moorter et al. 2013). The late flood was also characterised by high ambient temperatures, which likely reduced daytime movement and resulted in crepuscular peaks in displacement distance across both species. However, thermal responses of large herbivores are highly species‐specific and reflect interactions among morphology, physiology, behaviour and environmental conditions (Hetem et al. 2012; Fuller et al. 2016). Indeed, zebra showed a stronger reduction in movement with increasing maximum daily air temperature than tsessebe, indicating that thermal sensitivity cannot be predicted from digestive systems or body size alone (Hetem et al. 2012; Fuller et al. 2016). Cooler temperatures during the early flood season may have reduced thermal constraints on daytime movement and resulted in the higher peak movements at dawn and throughout the day, particularly for zebra.

Such flexibility may be related to their hindgut‐fermenting digestive strategy, which allows relatively continuous food intake without the periods of rumination required by ruminants (Schwarm et al. 2009; Owen‐Smith and Goodall 2014), although this mechanism cannot be distinguished from other species‐specific differences in our study. Reduced movement at night could additionally reflect responses to avoid nocturnal predation risk (Lima 1998; Packer et al. 2011; Cozzi et al. 2012; Veldhuis et al. 2020), however, predation risk was not quantified in our study.

The observed movement patterns are consistent with behavioural trade‐offs among resource acquisition and predator avoidance, highlighting the complex interactions among environmental conditions and species‐specific strategies that shape ungulate space use across the diel cycle. Our results indicate that behavioural flexibility in movement and activity timing plays a key role in mediating responses to changing seasonal environmental conditions. Both species adjusted their movement across seasons and the diel cycle but differed in the magnitude and timing of these responses. In particular, zebra movement responded more strongly to variation in maximum daily air temperature and vegetation greenness, whereas tsessebe generally used smaller areas and showed more localised movement patterns with greater tortuosity. Such differences may have important implications under ongoing climate change, as Africa is already experiencing rapid increases in temperature and climatic variability (Bedair et al. 2023; Engelbrecht et al. 2024). Rising temperatures and increasing aridity are expected to intensify thermal constraints and alter the spatial and temporal availability of forage and water, restricting foraging opportunities and potentially affecting species differently according to their capacity to adjust movement and resource use. Specialist species may be particularly vulnerable where environmental change reduces the availability of preferred resources, (Angert et al. 2011; Clavel et al. 2011). More broadly, because animal movement underpins ecosystem connectivity and the transfer of energy, biomass and genes, understanding how species‐specific physiological traits interact with environmental constraints is critical for anticipating climate‐driven changes and informing conservation and management strategies (Gable et al. 2023; Schmitz et al. 2023).

Because our comparison involved only two species and a relatively small number of individuals, differences between zebra and tsessebe cannot be attributed unequivocally to digestive systems, body size or forage specialisation. Future studies should integrate movement data with additional environmental variables, particularly fine‐scale surface water dynamics and habitat characteristics, as well as direct measures of physiological responses to better disentangle the mechanisms underlying seasonal movement patterns. Incorporating reproductive status and predator distributions would further improve our understanding of how ecological drivers shape movement decisions. Expanding these analyses to additional individuals, species and environmental conditions would help determine the generality of ungulate behavioural responses to environmental change.

## Author Contributions

**Zarah Schwan:** conceptualization (equal), data curation (supporting), formal analysis (lead), writing – original draft (lead). **W. Maartin Strauss:** conceptualization (equal), supervision (equal), writing – review and editing (equal). **Johannes Signer:** conceptualization (equal), supervision (equal), writing – review and editing (equal). **Robyn S. Hetem:** conceptualization (equal), funding acquisition (lead), supervision (equal), writing – review and editing (equal). **Marco Heurich:** writing – review and editing (supporting). **Gaseitsiwe Masunga:** supervision (equal), writing – review and editing (equal). **Alan M. Wilson:** data curation (lead), funding acquisition (equal). **Hattie L. A. Bartlam‐Brooks:** writing – review and editing (supporting). **Tatjana Y. Hubel:** writing – review and editing (supporting). **Emily Bennitt:** conceptualization (equal), data curation (equal), funding acquisition (equal), supervision (equal), writing – review and editing (equal).

## Funding

Zarah Schwan received funding for her PhD from the Southern African Science Service Centre for Climate Change and Adaptive Land Management; 01LG2099A. Original data collection was funded through Alan M. Wilson by H2020 European Research Council 323041; Engineering and Physical Sciences Research Council EP/H013016/1; Biotechnology and Biological Sciences Research Council BB/J018007/1.

## Conflicts of Interest

The authors declare no conflicts of interest.

## Supporting information


**Table S1:** Model selection table for of 24 h ranges (100% Minimum Convex Polygons, based on 5 min GPS locations) of plains zebra ( *Equus quagga* , *n* = 16) and tsessebe (*Damaliscus lunatus, n* = 8) across three seasons (early flood, late flood and rainy season) in the Okavango Delta, northern Botswana from 2014 to 2016 based on Alkaike's Information Criterion corrected for small sample size (AICc). Linear mixed effects models were fitted using log‐transformed MCP area as the response variable. Fixed effects included species, season, maximum daily air temperature, mean daily Enhanced Vegetation Index (EVI) and interactions between species and season, species and maximum daily air temperature and species and EVI, while individual identity was included as a random intercept in all models to account for repeated measurements of the same individuals and was therefore not considered during model selection. Models are ranked according to their AICc values. The table shows which fixed effects and interactions are included in each model (+), the number of parameters (df), AICc, the difference in AICc relative to the best model (delta AICc) and weight.
**Table S2:** Statistical results of Tukey‐adjusted post hoc comparisons testing interspecific seasonal differences in 24 h range sizes (100% MCP; km2) of plains zebra and tsessebe in the Okavango Delta, northern Botswana from 2014 to 2016. Estimates are presented on the log‐transformed scale of the fitted linear mixed‐effects model. Positive values indicate higher 24 h range sizes in the first‐listed species, whereas negative values indicate higher values in the second‐listed species. Significant results (*p* ≤ 0.05) are in bold.
**Figure S1:** Predicted daily 24 h minimum convex polygon (MCP) range size (km2) of plains zebra (blue; *n* = 16) and tsessebe (orange; *n* = 8) across the observed range of enhanced vegetation index (EVI) for the rainy, early flood and late flood seasons in the Okavango Delta, northern Botswana (2014–2016). Predictions were derived from the linear mixed‐effects model including species, season, maximum daily air temperature, EVI, the interaction between species and season and the interaction between species and maximum daily air temperature, with individual ID included as a random effect. Shaded areas represent 95% confidence intervals around the predicted values.
**Table S3:** Model selection table for 24 h displacement (summed distance moved between 5 min locations from sunrise to sunrise) of plains zebra (Equus quagga, *n* = 16) and tsessebe (Damaliscus lunatus, *n* = 8) across three seasons (early flood, late flood and rainy season) in the Okavango Delta, northern Botswana from 2014 to 2016 on Alkaike's Information Criterion corrected for small sample size (AICc). Linear mixed effect models were fitted using log‐transformed daily displacement as the response variable. Candidate fixed effects included species, season, maximum daily air temperature, mean daily Enhanced Vegetation Index (EVI) and their interactions as specified for each candidate model, while individual identity was included as a random intercept in all candidate models to account for repeated measurements within individuals and was therefore held constant during model selection. Models are ranked according to their AICc values. The table shows which fixed effect and interactions are included in each model (+), the number of parameters (df), AICc, the difference in AICc relative to the best model (delta AICc) and model weights.
**Table S4:** Interspecific differences between the mean 24 h displacement (m) of plains zebra (Equus quagga, *n* = 16) and tsessebe (Damaliscus lunatus, *n* = 8) during three seasons in north eastern Botswana: early flood season (May to July), late flood season (August to October) and rainy season (November to April). Values are given as estimated marginal means (back transformed to m) from the linear mixed model, as well as the z ratio calculated for the different seasons and associated *p*‐value representing the interspecific comparison between zebra and tsessebe within each season. Significant results (*p* ≤ 0.05) are in bold.
**Table S5:** Model selection table four hourly time block displacement of plains zebra (Equus quagga, *n* = 16) and tsessebe (Damaliscus lunatus, *n* = 8) across three seasons (early flood, late flood and rainy season) in the Okavango Delta, northern Botswana from 2014 to 2016, based on Alkaike's Information Criterion corrected for small sample size (AICc). Linear mixed effect models were fitted using log‐transformed four hourly time block displacement as the response variable. Candidate models included species, season, time block and their interactions up to the three‐way interaction, while individual identity was included as a random intercept to account for repeated measurements within individuals and was therefore held constant during model selection. Candidate models additionally evaluated the effects of maximum daily air temperature, mean daily Enhanced Vegetation Index (EVI) and species‐specific responses to these environmental variables. Models are ranked according to their AICc values. The table shows which fixed effect and interactions are included in each model (+), the number of parameters (df), AICc, the difference in AICc relative to the best model (delta AICc) and model weights.
**Table S6:** Intraspecific differences of the mean four hourly displacement across the seasons and time blocks of plains zebra (Equus quagga, *n* = 16) and tsessebe (Damaliscus lunatus, *n* = 8) during three seasons in north eastern Botswana: early flood season (May to July), late flood season (August to October) and rainy season (November to April). Values are given as estimated marginal means (back transformed to m) from the linear mixed model, as well as the z ratio and *p*‐value calculated for the different seasons usage and interspecific differences between zebra and tsessebe. Significant results (*p* ≤ 0.05) are in bold.
**Table S7:** Intraspecific differences of the mean four hourly displacement of the time blocks of plains zebra (Equus quagga, *n* = 16) and tsessebe (Damaliscus lunatus, *n* = 8) during three seasons in north eastern Botswana: early flood season (May to July), late flood season (August to October) and rainy season (November to April). Values are given as estimated marginal means (back transformed to km2) from the linear mixed model, as well as the z ratio calculated for the different seasons usage and interspecific differences between zebra and tsessebe. Significant results (*p* ≤ 0.05) are in bold.
**Table S8:** Model selection of movement tortuosity of plains zebra (Equus quagga, *n* = 16) and tsessebe (Damaliscus lunatus, *n* = 8) across three seasons (early flood, late flood and rainy season) and six four‐hour time blocks in the Okavango Delta, northern Botswana, from 2014 to 2016, based on Akaike's Information Criterion corrected for small sample size (AICc). Linear mixed‐effects models were fitted with the 4‐h tortuosity index as the response variable. Candidate fixed effects included species, season, time block, maximum daily air temperature, Enhanced Vegetation Index (EVI), the three‐way interaction among species, season and time block and interactions between species and maximum daily air temperature and between species and EVI. Individual identity was included as a random intercept to account for repeated measurements within individuals. Models are ranked according to their AICc values. The table shows which fixed effects and interactions are included in each model (+), the number of parameters (df), log‐likelihood (logLik), AICc, the difference in AICc relative to the best‐supported model (ΔAICc) and Akaike weight (wAIC).
**Table S9:** Intraspecific differences of tortuosity across the seasons and time blocks of plains zebra (Equus quagga, *n* = 16) and tsessebe (Damaliscus lunatus, *n* = 8) during three seasons in north eastern Botswana: early flood season (May to July), late flood season (August to October) and rainy season (November to April) and six time blocks (Dawn, Morning, Midday, Dusk, Early night and Late night). Values are given as estimated marginal means from the linear mixed model, as well as the z ratio calculated for the different seasons usage and interspecific differences between zebra and tsessebe. Significant results (*p* ≤ 0.05) are in bold.
**Table S10:** Intraspecific differences in movement tortuosity across six time blocks in plains zebra (Equus quagga, *N* = 16) and tsessebe (Damaliscus lunatus, *N* = 8) during three seasons in the Okavango Delta, northern Botswana: early flood season (May to July), late flood season (August to October) and rainy season (November to April). Time blocks were defined as dawn, morning, midday, dusk, early night and late night. Values represent pairwise contrasts of estimated marginal means from the linear mixed‐effects model, with estimates, standard errors (SE), z‐ratios and Tukey‐adjusted *p*‐values reported for comparisons among time blocks within each species and season. Significant results (*p* ≤ 0.05) are in bold.

## Data Availability

Data are available through the Dryad Digital Repository, https://doi.org/10.5061/dryad.6t1g1jxdj.
