# Supplementary material for: Balancing Food, Heat and Predation Risk: Seasonal and Diel Movement Strategies of African Herbivores
Source: Ecol Evol. 2026 Oct 16;16(10):e74498. doi: 10.1002/ece3.74498 (PMC13675114; doi:10.1002/ece3.74498)
Supplement: Supplementary file 1 [file ECE3-16-e74498-s001.docx]

**Supplementary material**

Table S1: Model selection table for of 24h ranges (100 % Minimum Convex Polygons, based on 5 min GPS locations) of plains zebra (*Equus quagga*, n = 16) and tsessebe (*Damaliscus lunatus,* n = 8) across three seasons (early flood, late flood and rainy season) in the Okavango Delta, northern Botswana from 2014-2016 based on Alkaike's Information Criterion corrected for small sample size (AICc). Linear mixed effects models were fitted using log-transformed MCP area as the response variable. Fixed effects included species, season, maximum daily air temperature, mean daily Enhanced Vegetation Index (EVI), and interactions between species and season, species and maximum daily air temperature, and species and EVI, while individual identity was included as a random intercept in all models to account for repeated measurements of the same individuals and was therefore not considered during model selection. Models are ranked according to their AICc values. The table shows which fixed effects and interactions are included in each model (+), the number of parameters (df), AICc, the difference in AICc relative to the best model (delta AICc) and weight.

| Intercept | season | Species | Temp_max | EVI | Season:Species | Species:temp_max | Species:EVI | df | logLik | AICc | Delta | Weight |
| --- | --- | --- | --- | --- | --- | --- | --- | --- | --- | --- | --- | --- |
| -1.11 | + | + | + | + | + | + |  | 11 | -14,551.20 | 29124.44 | 0.00 | 0.567 |
| -1.08 | + | + | + | + | + | + | + | 12 | -14550.47 | 29124.98 | 0.54 | 0.432 |
| -1.10 | + | + | + | + | + |  |  | 10 | -14559.67 | 29139.37 | 14.93 | 0 |
| -1.06 | + | + |  |  | + |  |  | 8 | -14578.53 | 29173.08 | 48.65 | 0 |
| -1.22 |  | + | + | + |  | + | + | 8 | -14598.07 | 29212.15 | 87.72 | 0 |

Table S2: *Statistical results of Tukey-adjusted post hoc comparisons testing interspecific seasonal differences in 24h range sizes (100% MCP; km²) of plains zebra and tsessebe in the Okavango Delta, northern Botswana from 2014 - 2016. Estimates are presented on the log-transformed scale of the fitted linear mixed-effects model. Positive values indicate higher 24h range sizes in the first-listed species, whereas negative values indicate higher values in the second-listed species. Significant results (p ≤ 0.05) are in bold.*

| Contrast | Season | Estimate | SE |
| --- | --- | --- | --- |
| Tsessebe - Zebra | Rainy season | **-0.925** | 0.428 |
| Tsessebe - Zebra | Early flood season | **-1.502** | 0.437 |
| Tsessebe - Zebra | Late flood season | -0.277 | 0.435 |

*Figure S1: Predicted daily 24h minimum convex polygon (MCP) range size (km2) of plains zebra (blue; n = 16) and tsessebe (orange; n = 8) across the observed range of enhanced vegetation index (EVI) for the rainy, early flood and late flood seasons in the Okavango Delta, northern Botswana (2014-2016). Predictions were derived from the linear mixed-effects model including species, season, maximum daily air temperature, EVI, the interaction between species and season, and the interaction between species and maximum daily air temperature, with individual ID included as a random effect. Shaded areas represent 95% confidence intervals around the predicted values.*

Table S3: Model selection table for 24h displacement (summed distance moved between 5 min locations from sunrise to sunrise) of plains zebra (*Equus quagga*, n = 16) and tsessebe (*Damaliscus lunatus,* n= 8) across three seasons (early flood, late flood and rainy season) in the Okavango Delta, northern Botswana from 2014-2016 on Alkaike´s Information Criterion corrected for small sample size (AICc). Linear mixed effect models were fitted using log-transformed daily displacement as the response variable. Candidate fixed effects included species, season, maximum daily air temperature, mean daily Enhanced Vegetation Index (EVI), and their interactions as specified for each candidate model, while individual identity was included as a random intercept in all candidate models to account for repeated measurements within individuals and was therefore held constant during model selection. Models are ranked according to their AICc values. The table shows which fixed effect and interactions are included in each model (+), the number of parameters (df), AICc, the difference in AICc relative to the best model (delta AICc), and model weights.

| Intercept | season | Species | Temp_  max | EVI | season:  Species | Species:  Temperature | Species:  EVI | df | logLik | AICc | Delta | Weight |
| --- | --- | --- | --- | --- | --- | --- | --- | --- | --- | --- | --- | --- |
| 8.758020 | + | + | + | + | + | + | + | 12 | -8,050.42 | 16124.88 | 0.00 | 0.51 |
| 8.742963 | + | + | + | + | + | + |  | 11 | -8,051.48 | 16124.99 | 0.11 | 0.49 |
| 8.745056 | + | + | + | + | + |  |  | 10 | -8,061.81 | 16143.66 | 18.77 | 0.00 |
| 8.752010 | + | + |  |  | + |  |  | 8 | -8,071.94 | 16159.91 | 35.07 | 0.00 |
| 8.632863 |  | + | + | + |  | + | + | 8 | -8,120.37 | 16256.75 | 131.87 | 0.00 |

Table S4: Interspecific differences between the mean 24h displacement (m) of plains zebra (*Equus quagga*, n = 16) and tsessebe (*Damaliscus lunatus,* n = 8) during three seasons in north eastern Botswana: early flood season (May to July), late flood season (August to October), and rainy season (November to April). Values are given as estimated marginal means (back transformed to m) from the linear mixed model, as well as the z ratio calculated for the different seasons and associated p value representing the interspecific comparison between zebra and tsessebe within each season. Significant results (p ≤ 0.05) are in bold.

| Species | Season | Estimate (m) | SE |
| --- | --- | --- | --- |
| Tsessebe | Early Flood | **4576.81** | 667.74 |
| Zebra | Early Flood | **7853.44** | 821.27 |
| Tsessebe | Late Flood | 5153.24 | 746.77 |
| Zebra | Late Flood | 5576.06 | 585.63 |
| Tsessebe | Rainy | 6266.44 | 898.11 |
| Zebra | Rainy | 8165.87 | 831.14 |

Table S5: Model selection table four hourly time block displacement of plains zebra (*Equus quagga*, n = 16) and tsessebe (*Damaliscus lunatus,* n = 8) across three seasons (early flood, late flood and rainy season) in the Okavango Delta, northern Botswana from 2014-2016, based on Alkaike´s Information Criterion corrected for small sample size (AICc). Linear mixed effect models were fitted using log-transformed four hourly time block displacement as the response variable. Candidate models included species, season, time block and their interactions up to the three-way interaction, while individual identity was included as a random intercept to account for repeated measurements within individuals and was therefore held constant during model selection. Candidate models additionally evaluated the effects of maximum daily air temperature, mean daily Enhanced Vegetation Index (EVI), and species-specific responses to these environmental variables. Models are ranked according to their AICc values. The table shows which fixed effect and interactions are included in each model (+), the number of parameters (df), AICc, the difference in AICc relative to the best model (delta AICc), and model weights.

| Intercept | season | Species | Time  block | Temperature | EVI | Season:  Species | Season:  Time block | Species:  Time block | Season:  Species:  time block | Species:  Temperature | Species:  EVI | df | logLik | AICc | Delta | Weight |
| --- | --- | --- | --- | --- | --- | --- | --- | --- | --- | --- | --- | --- | --- | --- | --- | --- |
| 7.282352 | + | + | + | + | + | + | + | + | + | + | + | 42 | -57498.83 | 115081.7 | 0.00 | 0.9921 |
| 7.263684 | + | + | + | + | + | + | + | + | + | + |  | 41 | -57504.67 | 115091.4 | 9.67 | 0.0079 |
| 7.265828 | + | + | + | + | + | + | + | + | + |  |  | 40 | -57544.53 | 115169.1 | 87.39 | 0 |
| 7.274298 | + | + | + |  |  | + | + | + | + |  |  | 38 | -57565.86 | 115207.8 | 126.05 | 0 |

Table S6: Intraspecific differences of the mean four hourly displacement across the seasons and time blocks of plains zebra (*Equus quagga*, n = 16) and tsessebe (*Damaliscus lunatus, n* = 8) during three seasons in north eastern Botswana: early flood season (May to July), late flood season (August to October), and rainy season (November to April). Values are given as estimated marginal means (back transformed to m) from the linear mixed model, as well as the z ratio and p value calculated for the different seasons usage and interspecific differences between zebra and tsessebe. Significant results (p ≤ 0.05) are in bold.

| Contrast Seasons | Species | Time Block | Estimate | SE | Z.ratio | P.value |
| --- | --- | --- | --- | --- | --- | --- |
| Rainy - Early flood | Tsessebe | Dawn | **0.16** | 0.05 | 3.30 | 0.003 |
| Rainy - Late flood | Tsessebe | Dawn | **0.17** | 0.04 | 3.74 | <0.001 |
| Early flood - Late flood | Tsessebe | Dawn | 0.01 | 0.05 | 0.11 | 0.99 |
| Rainy - Early flood | Zebra | Dawn | **-0.25** | 0.04 | -6.31 | < 0.001 |
| Rainy - Late flood | Zebra | Dawn | **0.31** | 0.04 | 7.44 | < 0.001 |
| Early flood - Late flood | Zebra | Dawn | **0.56** | 0.05 | 11.86 | < 0.001 |
| Rainy - Early flood | Tsessebe | Morning | **0.37** | 0.05 | 7.59 | < 0.001 |
| Rainy - Late flood | Tsessebe | Morning | **0.31** | 0.04 | 6.99 | < 0.001 |
| Early flood - Late flood | Tsessebe | Morning | -0.06 | 0.05 | -1.11 | 0.51 |
| Rainy - Early flood | Zebra | Morning | **-0.14** | 0.04 | -3.53 | 0.001 |
| Rainy - Late flood | Zebra | Morning | **0.42** | 0.04 | 9.95 | < 0.001 |
| Early flood - Late flood | Zebra | Morning | **0.56** | 0.05 | 11.69 | < 0.001 |
| Rainy - Early flood | Tsessebe | Midday | **0.32** | 0.05 | 6.49 | < 0.001 |
| Rainy - Late flood | Tsessebe | Midday | **0.30** | 0.04 | 6.70 | < 0.001 |
| Early flood - Late flood | Tsessebe | Midday | -0.02 | 0.05 | -0.37 | 0.93 |
| Rainy - Early flood | Zebra | Midday | **0.21** | 0.04 | 5.37 | < 0.001 |
| Rainy - Late flood | Zebra | Midday | **0.49** | 0.04 | 11.88 | < 0.001 |
| Early flood - Late flood | Zebra | Midday | **0.28** | 0.05 | 5.87 | < 0.001 |
| Rainy - Early flood | Tsessebe | Dusk | **0.53** | 0.05 | 10.89 | < 0.001 |
| Rainy - Late flood | Tsessebe | Dusk | **0.17** | 0.04 | 3.75 | < 0.001 |
| Early flood - Late flood | Tsessebe | Dusk | **-0.37** | 0.05 | -7.15 | < 0.001 |
| Rainy - Early flood | Zebra | Dusk | **0.32** | 0.4 | 8.16 | < 0.001 |
| Rainy - Late flood | Zebra | Dusk | **0.30** | 0.04 | 7.26 | < 0.001 |
| Early flood - Late flood | Zebra | Dusk | -0.02 | 0.05 | -0.52 | 0.86 |
| Rainy - Early flood | Tsessebe | Early night | **0.16** | 0.05 | 3.226 | 0.004 |
| Rainy - Late flood | Tsessebe | Early night | **0.28** | 0.04 | 6.34 | < 0.001 |
| Early flood - Late flood | Tsessebe | Early night | **0.12** | 0.05 | 2.44 | 0.039 |
| Rainy - Early flood | Zebra | Early night | 0.01 | 0.04 | 0.36 | 0.930 |
| Rainy - Late flood | Zebra | Early night | **0.33** | 0.04 | 7.99 | < 0.001 |
| Early flood - Late flood | Zebra | Early night | **0.32** | 0.05 | 6.71 | < 0.001 |
| Rainy - Early flood | Tsessebe | Late night | **0.56** | 0.05 | 11.43 | < 0.001 |
| Rainy - Late flood | Tsessebe | Late night | **0.61** | 0.04 | 13.77 | < 0.001 |
| Early flood - Late flood | Tsessebe | Late night | 0.05 | 0.05 | 1.02 | 0.562 |
| Rainy - Early flood | Zebra | Late night | 0.06 | 0.04 | 1.47 | 0.304 |
| Rainy - Late flood | Zebra | Late night | **0.32** | 0.04 | 7.67 | < 0.001 |
| Early flood - Late flood | Zebra | Late night | **0.26** | 0.05 | 5.48 | < 0.001 |

Table S7: Intraspecific differences of the mean four hourly displacement of the time blocks of plains zebra (*Equus quagga*, n = 16) and tsessebe (*Damaliscus lunatus,* n = 8) during three seasons in north eastern Botswana: early flood season (May to July), late flood season (August to October), and rainy season (November to April). Values are given as estimated marginal means (back transformed to km²) from the linear mixed model, as well as the z ratio calculated for the different seasons usage and interspecific differences between zebra and tsessebe. Significant results (p ≤ 0.05) are in bold.

| Contrast | Species | season | estimate | SE | z.ratio | p.value |
| --- | --- | --- | --- | --- | --- | --- |
| Dawn - Morning | Tsessebe | Rainy | **0.54** | 0.04 | 15.18 | < 0.001 |
| Dawn - Midday | Tsessebe | Rainy | **0.31** | 0.04 | 8.70 | < 0.001 |
| Dawn - Dusk | Tsessebe | Rainy | **0.24** | 0.04 | 6.80 | < 0.001 |
| Dawn - Early night cool safe | Tsessebe | Rainy | **0.89** | 0.04 | 25.16 | < 0.001 |
| Dawn - Late night cold predator | Tsessebe | Rainy | **1.14** | 0.04 | 32.19 | < 0.001 |
| Morning - Midday | Tsessebe | Rainy | **-0.23** | 0.04 | -6.48 | < 0.001 |
| Morning - Dusk | Tsessebe | Rainy | **-0.3** | 0.04 | -8.36 | < 0.001 |
| Morning - Early night cool safe | Tsessebe | Rainy | **0.35** | 0.04 | 9.97 | < 0.001 |
| Morning - Late night cold predator | Tsessebe | Rainy | **0.6** | 0.04 | 17.01 | < 0.001 |
| Midday - Dusk | Tsessebe | Rainy | -0.07 | 0.04 | -1.89 | 0.41 |
| Midday - Early night cool safe | Tsessebe | Rainy | **0.58** | 0.04 | 16.45 | < 0.001 |
| Midday - Late night cold predator | Tsessebe | Rainy | **0.83** | 0.04 | 23.49 | < 0.001 |
| Dusk - Early night cool safe | Tsessebe | Rainy | **0.65** | 0.04 | 18.33 | < 0.001 |
| Dusk - Late night cold predator | Tsessebe | Rainy | **0.9** | 0.04 | 25.36 | < 0.001 |
| Early night cool safe  - Late night cold predator | Tsessebe | Rainy | **0.25** | 0.04 | 7.06 | < 0.001 |
| Dawn - Morning | Zebra | Rainy | **0.15** | 0.04 | 5.53 | < 0.001 |
| Dawn - Midday | Zebra | Rainy | 0.01 | 0.03 | 0.35 | 1.00 |
| Dawn - Dusk | Zebra | Rainy | **-0.10** | 0.03 | -3.87 | 0.002 |
| Dawn - Early night cool safe | Zebra | Rainy | **0.55** | 0.03 | 20.73 | < 0.001 |
| Dawn - Late night cold predator | Zebra | Rainy | **0.86** | 0.03 | 32.25 | < 0.001 |
| Morning - Midday | Zebra | Rainy | **-0.14** | 0.03 | -5.19 | < 0.001 |
| Morning - Dusk | Zebra | Rainy | **-0.25** | 0.03 | -9.40 | < 0.001 |
| Morning - Early night cool safe | Zebra | Rainy | **0.41** | 0.03 | 15.27 | < 0.001 |
| Morning - Late night cold predator | Zebra | Rainy | **0.71** | 0.03 | 26.91 | < 0.001 |
| Midday - Dusk | Zebra | Rainy | **-0.11** | 0.03 | -4.23 | < 0.001 |
| Midday - Early night cool safe | Zebra | Rainy | **0.54** | 0.03 | 20.43 | < 0.001 |
| Midday - Late night cold predator | Zebra | Rainy | **0.85** | 0.03 | 32.08 | < 0.001 |
| Dusk - Early night cool safe | Zebra | Rainy | **0.65** | 0.03 | 24.56 | < 0.001 |
| Dusk - Late night cold predator | Zebra | Rainy | **0.96** | 0.03 | 36.17 | < 0.001 |
| Early night cool safe -  Late night cold predator | Zebra | Rainy | **0.31** | 0.03 | 11.54 | < 0.001 |
| Dawn - Morning | Tsessebe | Early flood | **0.75** | 0.05 | 13.82 | < 0.001 |
| Dawn - Midday | Tsessebe | Early flood | **0.47** | 0.05 | 8.58 | < 0.001 |
| Dawn - Dusk | Tsessebe | Early flood | **0.61** | 0.05 | 11.32 | < 0.001 |
| Dawn - Early night cool safe | Tsessebe | Early flood | **0.89** | 0.05 | 16.42 | < 0.001 |
| Dawn - Late night cold predator | Tsessebe | Early flood | **1.54** | 0.05 | 28.42 | < 0.001 |
| Morning - Midday | Tsessebe | Early flood | **-0.28** | 0.05 | -5.21 | < 0.001 |
| Morning - Dusk | Tsessebe | Early flood | -0.14 | 0.05 | -2.50 | 0.124 |
| Morning - Early night cool safe | Tsessebe | Early flood | 0.14 | 0.05 | 2.59 | 0.10 |
| Morning - Late night cold predator | Tsessebe | Early flood | **0.79** | 0.05 | 14.61 | < 0.001 |
| Midday - Dusk | Tsessebe | Early flood | 0.15 | 0.05 | 2.71 | 0.07 |
| Midday - Early night cool safe | Tsessebe | Early flood | **0.42** | 0.05 | 7.79 | < 0.001 |
| Midday - Late night cold predator | Tsessebe | Early flood | **1.08** | 0.05 | 19.78 | < 0.001 |
| Dusk - Early night cool safe | Tsessebe | Early flood | **0.28** | 0.05 | 5.09 | < 0.001 |
| Dusk - Late night cold predator | Tsessebe | Early flood | **0.93** | 0.05 | 17.11 | < 0.001 |
| Early night cool safe -  Late night cold predator | Tsessebe | Early flood | **0.65** | 0.05 | 12.05 | < 0.001 |
| Dawn - Morning | Zebra | Early flood | **0.25** | 0.04 | 5.59 | < 0.001 |
| Dawn - Midday | Zebra | Early flood | **0.47** | 0.04 | 10.50 | < 0.001 |
| Dawn - Dusk | Zebra | Early flood | **0.47** | 0.04 | 10.50 | < 0.001 |
| Dawn - Early night cool safe | Zebra | Early flood | **0.81** | 0.04 | 18.24 | < 0.001 |
| Dawn - Late night cold predator | Zebra | Early flood | **1.17** | 0.04 | 26.05 | < 0.001 |
| Morning - Midday | Zebra | Early flood | **0.22** | 0.05 | 4.79 | < 0.001 |
| Morning - Dusk | Zebra | Early flood | **0.22** | 0.05 | 4.79 | < 0.001 |
| Morning - Early night cool safe | Zebra | Early flood | **0.56** | 0.04 | 12.46 | < 0.001 |
| Morning - Late night cold predator | Zebra | Early flood | **0.91** | 0.05 | 20.24 | < 0.001 |
| Midday - Dusk | Zebra | Early flood | -0.0004 | 0.05 | -0.01 | 1.00 |
| Midday - Early night cool safe | Zebra | Early flood | **0.34** | 0.05 | 7.62 | < 0.001 |
| Midday - Late night cold predator | Zebra | Early flood | **0.70** | 0.05 | 15.39 | < 0.001 |
| Dusk - Early night cool safe | Zebra | Early flood | **0.34** | 0.05 | 7.64 | < 0.001 |
| Dusk - Late night cold predator | Zebra | Early flood | **0.70** | 0.05 | 15.42 | < 0.001 |
| Early night cool safe -  Late night cold predator | Zebra | Early flood | **0.35** | 0.05 | 7.82 | < 0.001 |
| Dawn - Morning | Tsessebe | Late flood | **0.69** | 0.05 | 15.27 | < 0.001 |
| Dawn - Midday | Tsessebe | Late flood | **0.44** | 0.04 | 9.88 | < 0.001 |
| Dawn - Dusk | Tsessebe | Late flood | **0.24** | 0.04 | 5.43 | < 0.001 |
| Dawn - Early night cool safe | Tsessebe | Late flood | **1.01** | 0.04 | 22.66 | < 0.001 |
| Dawn - Late night cold predator | Tsessebe | Late flood | **1.59** | 0.04 | 35.72 | < 0.001 |
| Morning - Midday | Tsessebe | Late flood | **-0.24** | 0.05 | -5.46 | < 0.001 |
| Morning - Dusk | Tsessebe | Late flood | **-0.44** | 0.04 | -9.93 | < 0.001 |
| Morning - Early night cool safe | Tsessebe | Late flood | **0.32** | 0.04 | 7.19 | < 0.001 |
| Morning - Late night cold predator | Tsessebe | Late flood | **0.90** | 0.05 | 20.17 | < 0.001 |
| Midday - Dusk | Tsessebe | Late flood | **-0.20** | 0.04 | -4.49 | < 0.001 |
| Midday - Early night cool safe | Tsessebe | Late flood | **0.57** | 0.04 | 12.75 | < 0.001 |
| Midday - Late night cold predator | Tsessebe | Late flood | **1.15** | 0.04 | 25.83 | < 0.001 |
| Dusk - Early night cool safe | Tsessebe | Late flood | **0.77** | 0.04 | 17.29 | < 0.001 |
| Dusk - Late night cold predator | Tsessebe | Late flood | **1.35** | 0.04 | 30.40 | < 0.001 |
| Early night cool safe -  Late night cold predator | Tsessebe | Late flood | **0.58** | 0.04 | 13.12 | < 0.001 |
| Dawn - Morning | Zebra | Late flood | **0.25** | 0.05 | 5.33 | < 0.001 |
| Dawn - Midday | Zebra | Late flood | **0.19** | 0.05 | 4.05 | < 0.001 |
| Dawn - Dusk | Zebra | Late flood | -0.11 | 0.05 | -2.41 | 0.15 |
| Dawn - Early night cool safe | Zebra | Late flood | **0.57** | 0.05 | 12.21 | < 0.001 |
| Dawn - Late night cold predator | Zebra | Late flood | **0.87** | 0.05 | 18.47 | < 0.001 |
| Morning - Midday | Zebra | Late flood | -0.06 | 0.05 | -1.32 | 0.78 |
| Morning - Dusk | Zebra | Late flood | **-0.37** | 0.05 | -7.76 | < 0.001 |
| Morning - Early night cool safe | Zebra | Late flood | **0.32** | 0.05 | 6.80 | < 0.001 |
| Morning - Late night cold predator | Zebra | Late flood | **0.62** | 0.05 | 13.05 | < 0.001 |
| Midday - Dusk | Zebra | Late flood | **-0.30** | 0.05 | -6.50 | < 0.001 |
| Midday - Early night cool safe | Zebra | Late flood | **0.38** | 0.05 | 8.19 | < 0.001 |
| Midday - Late night cold predator | Zebra | Late flood | **0.68** | 0.05 | 14.49 | < 0.001 |
| Dusk - Early night cool safe | Zebra | Late flood | **0.69** | 0.05 | 14.70 | < 0.001 |
| Dusk - Late night cold predator | Zebra | Late flood | **0.98** | 0.05 | 20.99 | < 0.001 |
| Early night cool safe -  Late night cold predator | Zebra | Late flood | **0.30** | 0.05 | 6.32 | < 0.001 |

Table S8: Model selection of movement tortuosity of plains zebra (*Equus quagga*, n = 16) and tsessebe (*Damaliscus lunatus, n* = 8) across three seasons (early flood, late flood and rainy season) and six four-hour time blocks in the Okavango Delta, northern Botswana, from 2014–2016, based on Akaike's Information Criterion corrected for small sample size (AICc). Linear mixed-effects models were fitted with the four-hour tortuosity index as the response variable. Candidate fixed effects included species, season, time block, maximum daily air temperature, Enhanced Vegetation Index (EVI), the three-way interaction among species, season and time block, and interactions between species and maximum daily air temperature and between species and EVI. Individual identity was included as a random intercept to account for repeated measurements within individuals. Models are ranked according to their AICc values. The table shows which fixed effects and interactions are included in each model (+), the number of parameters (df), log-likelihood (logLik), AICc, the difference in AICc relative to the best-supported model (ΔAICc), and Akaike weight (wAIC).

| Intercept | season | Species | Time block | Temperature | EVI | season:Species | Season:time block | species: time block | Season: species. time block | Species: Temperature | Species:EVI | df | logLik | AICc | delta | weight |
| --- | --- | --- | --- | --- | --- | --- | --- | --- | --- | --- | --- | --- | --- | --- | --- | --- |
| 0.66985 | + | + | + | + | + | + | + | + | + | + | + | 42 | 5,602.44 | -11,120.79 | 0.0 | 0.992 |
| 0.6943 | + | + | + | + | + | + | + | + | + | + |  | 41 | 5,596.73 | --11,111.36 | 9.43 | 0.009 |
| 0.6943 | + | + | + | + | + | + | + | + | + |  |  | 40 | 5,591.43 | --11,102.77 | 18.02 | <0.001 |
| 0.7019 | + | + | + |  |  | + | + | + | + |  |  | 38 | 5,564.18 | -11,052.28 | 68.51 | <0.001 |

Table S9: Intraspecific differences of tortuosity across the seasons and time blocks of plains zebra (*Equus quagga*, n = 16) and tsessebe (*Damaliscus lunatus, n* = 8) during three seasons in north eastern Botswana: early flood season (May to July), late flood season (August to October), and rainy season (November to April) and six time blocks (Dawn, Morning, Midday, Dusk, Early night and Late night). Values are given as estimated marginal means from the linear mixed model, as well as the z ratio calculated for the different seasons usage and interspecific differences between zebra and tsessebe. Significant results (p ≤ 0.05) are in bold.

| Season contrast | Species | Time block | Estimate | SE |
| --- | --- | --- | --- | --- |
| Rainy- Early flood | Tsessebe | Late night | 0.000 | 0.011 |
| Rainy - Late flood | Tsessebe | Late night | **0.071** | 0.010 |
| Early flood- Late flood | Tsessebe | Late night | **0.071** | 0.011 |
| Rainy - Early flood | Zebra | Late night | **0.109** | 0.009 |
| Rainy - Late flood | Zebra | Late night | 0.021 | 0.010 |
| Early flood- Late flood | Zebra | Late night | **-0.088** | 0.011 |
| Rainy- Early flood | Tsessebe | Dawn | -0.012 | 0.011 |
| Rainy- Late flood | Tsessebe | Dawn | 0.018 | 0.010 |
| Early flood- Late flood | Tsessebe | Dawn | **0.030** | 0.011 |
| Rainy - Early flood | Zebra | Dawn | 0.006 | 0.009 |
| Rainy- Late flood | Zebra | Dawn | -0.005 | 0.010 |
| Early flood - Late flood | Zebra | Dawn | -0.011 | 0.011 |
| Rainy - Early flood | Tsessebe | Morning | 0.011 | 0.011 |
| Rainy- Late flood | Tsessebe | Morning | 0.010 | 0.010 |
| Early flood- Late flood | Tsessebe | Morning | -0.001 | 0.011 |
| Rainy- Early flood | Zebra | Morning | 0.002 | 0.009 |
| Rainy - Late flood | Zebra | Morning | 0.003 | 0.010 |
| Early flood - Late flood | Zebra | Morning | 0.001 | 0.012 |
| Rainy - Early flood | Tsessebe | Midday | **0.040** | 0.011 |
| Rainy- Late flood | Tsessebe | Midday | **0.070** | 0.010 |
| Early flood - Late flood | Tsessebe | Midday | **0.030** | 0.011 |
| Rainy - Early flood | Zebra | Midday | 0.020 | 0.010 |
| Rainy- Late flood | Zebra | Midday | 0.002 | 0.010 |
| Early flood - Late flood | Zebra | Midday | -0.018 | 0.012 |
| Rainy - Early flood | Tsessebe | Dusk | 0.013 | 0.011 |
| Rainy - Late flood | Tsessebe | Dusk | **0.055** | 0.010 |
| Early flood- Late flood | Tsessebe | Dusk | **0.042** | 0.011 |
| Rainy - Early flood | Zebra | Dusk | 0.006 | 0.010 |
| Rainy - Late flood | Zebra | Dusk | -0.009 | 0.009 |
| Early flood - Late flood | Zebra | Dusk | -0.016 | 0.011 |
| Rainy - Early flood | Tsessebe | Early night | 0.016 | 0.011 |
| Rainy- Late flood | Tsessebe | Early night | **0.042** | 0.010 |
| Early flood- Late flood | Tsessebe | Early night | 0.026 | 0.011 |
| Rainy- Early flood | Zebra | Early night | 0.009 | 0.009 |
| Rainy - Late flood | Zebra | Early night | **-0.028** | 0.009 |
| Early flood- Late flood | Zebra | Early night | **-0.037** | 0.011 |

Table S10: Intraspecific differences in movement tortuosity across six time blocks in plains zebra (*Equus quagga*, N= 16) and tsessebe (*Damaliscus lunatus,* N= 8) during three seasons in the Okavango Delta, northern Botswana: early flood season (May to July), late flood season (August to October), and rainy season (November to April). Time blocks were defined as dawn, morning, midday, dusk, early night, and late night. Values represent pairwise contrasts of estimated marginal means from the linear mixed-effects model, with estimates, standard errors (SE), z-ratios, and Tukey-adjusted p-values reported for comparisons among time blocks within each species and season. Significant results (p ≤ 0.05) are in bold.

| Time-block contrast | Species | Season | Estimate | SE |
| --- | --- | --- | --- | --- |
| Late night - Dawn | Tsessebe | Rainy | **0.044** | 0.008 |
| Late night - Morning | Tsessebe | Rainy | **0.077** | 0.008 |
| Late night - Midday | Tsessebe | Rainy | **0.137** | 0.008 |
| Late night - Dusk | Tsessebe | Rainy | **0.146** | 0.008 |
| Late night - Early night | Tsessebe | Rainy | **0.086** | 0.008 |
| Dawn - Morning | Tsessebe | Rainy | **0.033** | 0.008 |
| Dawn - Midday | Tsessebe | Rainy | **0.093** | 0.008 |
| Dawn - Dusk | Tsessebe | Rainy | **0.102** | 0.008 |
| Dawn - Early night | Tsessebe | Rainy | **0.042** | 0.008 |
| Morning - Midday | Tsessebe | Rainy | **0.060** | 0.008 |
| Morning - Dusk | Tsessebe | Rainy | **0.069** | 0.008 |
| Morning - Early night | Tsessebe | Rainy | 0.009 | 0.008 |
| Midday - Dusk | Tsessebe | Rainy | 0.009 | 0.008 |
| Midday - Early night | Tsessebe | Rainy | **-0.051** | 0.008 |
| Dusk - Early night | Tsessebe | Rainy | **-0.060** | 0.008 |
| Late night - Dawn | Zebra | Rainy | **0.050** | 0.006 |
| Late night - Morning | Zebra | Rainy | **0.107** | 0.006 |
| Late night - Midday | Zebra | Rainy | **0.115** | 0.006 |
| Late night - Dusk | Zebra | Rainy | **0.162** | 0.006 |
| Late night - Early night | Zebra | Rainy | **0.123** | 0.006 |
| Dawn - Morning | Zebra | Rainy | **0.057** | 0.006 |
| Dawn - Midday | Zebra | Rainy | **0.065** | 0.006 |
| Dawn - Dusk | Zebra | Rainy | **0.112** | 0.006 |
| Dawn - Early night | Zebra | Rainy | **0.073** | 0.006 |
| Morning - Midday | Zebra | Rainy | 0.008 | 0.006 |
| Morning - Dusk | Zebra | Rainy | **0.054** | 0.006 |
| Morning - Early night | Zebra | Rainy | 0.016 | 0.006 |
| Midday - Dusk | Zebra | Rainy | **0.047** | 0.006 |
| Midday - Early night | Zebra | Rainy | 0.008 | 0.006 |
| Dusk - Early night | Zebra | Rainy | **-0.038** | 0.006 |
| Late night - Dawn | Tsessebe | Early flood | 0.032 | 0.012 |
| Late night - Morning | Tsessebe | Early flood | **0.088** | 0.012 |
| Late night - Midday | Tsessebe | Early flood | **0.177** | 0.012 |
| Late night - Dusk | Tsessebe | Early flood | **0.159** | 0.012 |
| Late night - Early night | Tsessebe | Early flood | **0.103** | 0.012 |
| Dawn - Morning | Tsessebe | Early flood | **0.056** | 0.012 |
| Dawn - Midday | Tsessebe | Early flood | **0.145** | 0.012 |
| Dawn - Dusk | Tsessebe | Early flood | **0.127** | 0.012 |
| Dawn - Early night | Tsessebe | Early flood | **0.070** | 0.012 |
| Morning - Midday | Tsessebe | Early flood | **0.089** | 0.012 |
| Morning - Dusk | Tsessebe | Early flood | **0.072** | 0.012 |
| Morning - Early night | Tsessebe | Early flood | 0.015 | 0.012 |
| Midday - Dusk | Tsessebe | Early flood | -0.018 | 0.012 |
| Midday - Early night | Tsessebe | Early flood | **-0.074** | 0.012 |
| Dusk - Early night | Tsessebe | Early flood | **-0.057** | 0.012 |
| Late night - Dawn | Zebra | Early flood | **-0.052** | 0.010 |
| Late night - Morning | Zebra | Early flood | 0.000 | 0.010 |
| Late night - Midday | Zebra | Early flood | 0.026 | 0.011 |
| Late night - Dusk | Zebra | Early flood | **0.059** | 0.011 |
| Late night - Early night | Zebra | Early flood | 0.024 | 0.010 |
| Dawn - Morning | Zebra | Early flood | **0.053** | 0.010 |
| Dawn - Midday | Zebra | Early flood | **0.078** | 0.011 |
| Dawn - Dusk | Zebra | Early flood | **0.111** | 0.011 |
| Dawn - Early night | Zebra | Early flood | **0.076** | 0.010 |
| Morning - Midday | Zebra | Early flood | 0.026 | 0.011 |
| Morning - Dusk | Zebra | Early flood | **0.059** | 0.011 |
| Morning - Early night | Zebra | Early flood | 0.023 | 0.010 |
| Midday - Dusk | Zebra | Early flood | 0.033 | 0.012 |
| Midday - Early night | Zebra | Early flood | -0.002 | 0.011 |
| Dusk - Early night | Zebra | Early flood | **-0.035** | 0.011 |
| Late night - Dawn | Tsessebe | Late flood | -0.009 | 0.010 |
| Late night - Morning | Tsessebe | Late flood | 0.016 | 0.010 |
| Late night - Midday | Tsessebe | Late flood | **0.136** | 0.010 |
| Late night - Dusk | Tsessebe | Late flood | **0.130** | 0.010 |
| Late night - Early night | Tsessebe | Late flood | **0.057** | 0.010 |
| Dawn - Morning | Tsessebe | Late flood | 0.025 | 0.010 |
| Dawn - Midday | Tsessebe | Late flood | **0.145** | 0.010 |
| Dawn - Dusk | Tsessebe | Late flood | **0.139** | 0.010 |
| Dawn - Early night | Tsessebe | Late flood | **0.066** | 0.010 |
| Morning - Midday | Tsessebe | Late flood | **0.120** | 0.010 |
| Morning - Dusk | Tsessebe | Late flood | **0.114** | 0.010 |
| Morning - Early night | Tsessebe | Late flood | **0.041** | 0.010 |
| Midday - Dusk | Tsessebe | Late flood | -0.006 | 0.010 |
| Midday - Early night | Tsessebe | Late flood | **-0.079** | 0.010 |
| Dusk - Early night | Tsessebe | Late flood | **-0.073** | 0.010 |
| Late night - Dawn | Zebra | Late flood | 0.025 | 0.011 |
| Late night - Morning | Zebra | Late flood | **0.089** | 0.012 |
| Late night - Midday | Zebra | Late flood | **0.096** | 0.011 |
| Late night - Dusk | Zebra | Late flood | **0.131** | 0.011 |
| Late night - Early night | Zebra | Late flood | **0.074** | 0.011 |
| Dawn - Morning | Zebra | Late flood | **0.065** | 0.012 |
| Dawn - Midday | Zebra | Late flood | **0.072** | 0.011 |
| Dawn - Dusk | Zebra | Late flood | **0.107** | 0.011 |
| Dawn - Early night | Zebra | Late flood | **0.050** | 0.011 |
| Morning - Midday | Zebra | Late flood | 0.007 | 0.012 |
| Morning - Dusk | Zebra | Late flood | **0.042** | 0.011 |
| Morning - Early night | Zebra | Late flood | -0.015 | 0.011 |
| Midday - Dusk | Zebra | Late flood | **0.035** | 0.011 |
| Midday - Early night | Zebra | Late flood | -0.022 | 0.011 |
| Dusk - Early night | Zebra | Late flood | **-0.057** | 0.010 |
